# Terminal ballistic analysis of impact fractures reveals the use of spearthrower 31 ky ago at Maisières-Canal, Belgium

**DOI:** 10.1038/s41598-023-45554-w

**Published:** 2023-10-25

**Authors:** Justin Coppe, Noora Taipale, Veerle Rots

**Affiliations:** 1 TraceoLab/Prehistory, University of Liège, Place du 20-Août 7 (Bât. A4), 4000 Liège, Belgium; 2 F.R.S.-FNRS, University of Liège, Liège, Belgium

**Keywords:** Anthropology, Archaeology, Evolution

## Abstract

The emergence of hunting technology in the deep past fundamentally shaped the subsistence strategies of early human populations. Hence knowing when different weapons were first introduced is important for understanding our evolutionary trajectory. The timing of the adoption of long-range weaponry remains heavily debated because preserved organic weapon components are extremely rare in the Paleolithic record and stone points are difficult to attribute reliably to weapon delivery methods without supporting organic evidence. Here, we use a refined use-wear approach to demonstrate that spearthrower was used for launching projectiles armed with tanged flint points at Maisières-Canal (Belgium) 31,000 years ago. The novelty of our approach lies in the combination of impact fracture data with terminal ballistic analysis of the mechanical stress suffered by a stone armature on impact. This stress is distinct for each weapon and visible archaeologically as fracture proportions on assemblage scale. Our reference dataset derives from a sequential experimental program that addressed individually each key parameter affecting fracture formation and successfully reproduced the archaeological fracture signal. The close match between the archaeological sample and the experimental spearthrower set extends the timeline of spearthrower use by over 10,000 years and represents the earliest reliable trace-based evidence for the utilization of long-distance weaponry in prehistoric hunting.

## Introduction

Humans have been hunter-gatherers for most of our past, but we still lack knowledge about what our prehistoric ancestors gathered and how they hunted because of the near lack of organic preservation at Paleolithic sites. Of these two modes of subsistence, hunting tends to be better visible archaeologically because it can leave behind durable stone and bone implements that once served as weapon components. Four weapon systems—thrusted spears, thrown spears, the spearthrower, and the bow—are assumed to have existed in the Stone Age, but the timing of their invention and their possible co-existence remain debated. Self-pointed wooden spears or their fragments have been found at Clacton-on-Sea, dated to around 400 ka^1, 2^ and at Schöningen, dated to c. 300 ka^3, 4^. The Schöningen weapons have been attributed to *Homo heidelbergensis* or early Neanderthals^4, 5^. Neanderthal use of wooden spears is further supported by direct evidence from Lehringen, where several spear fragments were found in association with forest elephant remains^6^ and by experimental replication of hunting lesions documented on faunal remains from Neumark-Nord^7^, both sites dated to MIS 5e. Whether Neanderthals used spears as thrusted or thrown weapons, or both, is under debate^8–20^. The question whether they had other weaponry at their disposal has generally provoked more opinions than hard evidence^10^, but evidence for diversity of early hunting toolkits is currently emerging^21^.

The ethnographic record shows that throwing and thrusting spears were frequently used in combination at close range in disadvantage hunting^19, 22–24^, which refers to trapping the prey in a specific location by collective action, by exploiting landscape features, or both. The bow and the spearthrower can be used in similar contexts, but their extended range makes the hunter less dependent on disadvantaging the prey and thus facilitates solitary hunting as well as capturing prey in open environments void of natural traps^8, 11, 22, 25, 26^. These advantages do not necessarily translate into higher returns^24, 27^ but rather have major implications for hunting strategies, with consequences for the overall organization of activities within a social group^28, 29^.

In contrast with thrusted or hand-cast spears that were used by Neanderthals and modern humans alike^2^, archaeological literature systematically associates long-range weaponry—the spearthrower and the bow—with anatomically modern *Homo sapiens*. An explicit narrative exists about modern humans possessing superior weaponry that would have helped them disperse out of Africa and outcompete Neanderthals in Europe^16, 17, 30–33^. Certain influential works have put forward a hypothesis that the origins of long-range weaponry could lie in the African Middle Stone Age (MSA) where assemblages contain small-sized points^34^, an idea echoed by later studies^35–39^. Yet, the oldest direct evidence are the preserved arrows from Stellmoor (northern Germany) that have been attributed to the Younger Dryas^40^ and dated to around 12–11 ka cal BP^41^. The oldest definite spearthrower hooks are lower Magdalenian and date to c. 20 ka cal BP^42^.

## Emergence of long-range weaponry: current perspectives and methodological challenges

Several syntheses imply or directly state that there is a consensus among researchers that long-range weapons were known to modern humans by the beginning of the Upper Paleolithic^43–46^. Their first invention has been frequently placed between 100 and 45 ka BP^16, 35, 36, 39, 47, 48^, a period for which no direct evidence is currently available. The archaeological record presents a gap of c. 100,000 years between the youngest Middle Paleolithic preserved wooden spears^6^ and the oldest preserved Upper Paleolithic spearthrower hooks^42^. Understanding developments during this important period necessitates methodologies that make use of remains that are more abundantly available than preserved organic weapon elements. Functional analysis methods that focus on macroscopic and microscopic traces preserved on lithic and organic implements have been repeatedly called for aid^16, 49^, but their application for identifying weapon delivery systems is not without complications.

Early African origin of bow and arrow is one of the cornerstones of the present view of long-range weaponry as a uniquely modern human feature. Both lithic and organic artefacts from southern African Middle Stone Age contexts have been interpreted as arrowheads based on use damage e.g.^36–38^, with some of the lithic evidence later challenged^50–53^. The arguments for attributing the proposed stone and bone weapon tips to bow and arrow are exclusively size-related^36, 38^. In projectile studies, the size argument has been formalized and extended to various point morphologies with the introduction of a particular measure, the tip cross-sectional area (TCSA)^54^. This approach has become tremendously popular and continues to be applied^11, 12, 39, 45, 49, 55–69^ even though its reliability can be questioned in the light of ethnographic data as well as experimental observations (SI Text 1.1). Therefore, while remaining a relevant hypothesis, the presence of long-range weaponry in southern Africa in the Middle Stone Age remains to be proved. The latest claim for the early use of bow in Europe, while presenting use-wear on the archaeological sample, also belongs to this group of studies that use the small size of the (damaged) pieces as the sole argument for bow and arrow^45^.

In Ethiopia where obsidian points are common, researchers have recently explored the analysis of microscopic features on impact fracture surfaces as a supplementary means to identify weapon systems^39, 70^, but see^71, 72^. The method, developed previously by Hutchings through experimental work, is based on the measurement of Wallner lines and fracture wings observable on fracture surfaces to determine the speed of crack propagation^73–75^. The main challenge in applying this analysis on archaeological projectile points on a global scale is that the relevant features occur mostly in glassy materials like obsidian and quartz^76^. Sahle and Brooks who sought to use fracture wings as an additional line of evidence for complex weaponry at 80–100 kya in Ethiopia were confronted by a limited number of fractures suitable for this kind of analysis due to the surface preservation of the archaeological obsidian^39^. Their proposition that spearthrowers may have been used at Aduma currently relies solely on metric data. An earlier attempt to apply similar methodology to older points from the Gademotta formation^70^, for critique, see^71, 72^ concluded that the archaeological specimens were in the range of hand-cast spears rather than darts or bows. Therefore, while Wallner lines and fracture wings may well present a complementary way to investigate impact fracture propagation and its relationship with weapon ballistics in future projectile studies, the contribution of this line of research to the debate on the emergence of long-range projectile weaponry is at present limited.

In the study of projectile origins, thus far the most fruitful and widely applicable approach has been the one focused on macroscopic fracture patterns and associated microscopic features. This line of inquiry has been developed through experiments to investigate the link between weapon systems and macroscopic impact fracture characteristics^75, 77–82^ (SI Text S1.2) and has been pursued by researchers working on Early Upper Paleolithic material related to modern human dispersals in East Asia, Europe, and the Middle East^47, 48, 83^. The experiments^75, 77–82^ have yielded significant discoveries and established a link between impact velocity (or kinetic energy) and the intensity of impact damage (fracture frequency and size) in artificial experimental settings aimed to control key variables^75, 78–80^. Archaeological applications have targeted Early Upper Paleolithic assemblages in Japan where bow use has been argued for^47^, the Uluzzian in Italy that has been said to bear evidence for spearthrower or bow^48^, and the earliest Upper Paleolithic in the Levant where long-range weapons are claimed to have existed^83^. These results are intriguing, but a major issue remains in matching the experimental and archaeological datasets. It is related to the difficulty of reproducing the impact stress generated by prehistoric weapons using the machine-assisted experimental setups the authors opted for. In our view, the experiments carried out so far have largely addressed projectile velocity or kinetic energy, overlooking other relevant variables controlling fracture formation, such as the angle of incidence (SI Text S1.2). Due to these omissions, there are not yet experimental reference collections that would permit identifying prehistoric weapon systems reliably by analyzing macroscopic fractures on stone points.

We propose here a revised macrofracture approach that is based on the principle that each prehistoric weapon shows a different terminal ballistic behavior due to contrasting values of angle of incidence, kinetic energy, and other variables influencing the nature of contact between the projectile and its target. These differences translate into distinct mechanical stresses to which the stone point is subjected. The stress conditions influence fracture formation in a selective manner, promoting or hindering the occurrence of different categories of fractures. Each weapon delivery system thus leaves a macrofracture signature visible in the relative proportions of fractures on impact-damaged projectile points when samples are sufficiently large^84^. The potential of relative fracture frequencies for prehistoric weapon identification has been tested previously, but researchers have not yet succeeded in reaching satisfactory archaeological results (SI Text S1.3). Our experiment-based fracture mechanical method relies on a large set of data on experimental impact fractures and on the ballistic analysis of replicas of prehistoric weapons. Its first application on archaeological material allowed us to identify the use of spearthrower around 31,000 years ago at the Early Upper Paleolithic site of Maisières-Canal (Belgium).

## Archaeological context

Maisières-Canal is a reference site for the Early Upper Paleolithic of north-west Europe and one of the few Early Upper Paleolithic open-air sites known from the region. It is located in Belgium near the town of Mons, on the northern edge of the alluvial plain of the Haine. The occupation layers that had previously been concealed by ground water were first discovered in 1966 by G. Bois d’Enghien during the works for widening the Canal du Centre. A rescue excavation was carried out the same year by a team led by J. de Heinzelin from the Royal Belgian Institute of Natural Sciences^85–88^.

The remains of the occupation were remarkably well preserved in situ within a sedimentary sequence consisting of fluviatile deposits and humic horizons. The largest concentration of remains that is the focus of the present study was excavated over an area of 95 m^2^ and yielded more than 34,000 lithics together with organic artefacts and their manufacturing waste, faunal remains, and burnt bone and stone indicative of hearths. The site has been interpreted as having a single main Early Upper Paleolithic occupation layer that shows limited vertical displacement of finds. Thirteen radiocarbon dates consistently place the occupation to around 28,000 BP/31,000 cal BP^85, 86, 89–95^. The date 27,950 ± 170 BP obtained on a cut-marked reindeer bone (OxA-18007) has been particularly favoured as representing the age of the layer^91^.

The calibration of radiocarbon ages suggests that the occupation took place around Dansgaard-Oeschger event 5^96, 97^. Sedimentological analysis indicates that the archaeological layer formed during moderately cold conditions, similar to the present-day climate between southern Scandinavia and northern Siberia^85^. The environment at Maisières-Canal has been reconstructed as steppe or tundra with a marshland component^85, 87, 98^, with trees such as pine, alder, willow, and birch probably present in sheltered areas in the region^85^. The archeozoological study documented a variety of species typical of a cold, humid environment, including reindeer (MNI = 2), horse (MNI = 1), bear (MNI = 1), aurochs/bison (MNI = 1), and cervids (MNI = 1), as well as small fauna, such as hare (MNI = 8), fox (MNI = 6), and various birds (MNI = 17)^87, 99^. The MNI counts are overall brought down by the high proportion of heavily fragmented and burnt bone in the collection^87, 100^. Evidence on seasonality is currently limited, but juvenile reindeer remains and avian fauna point to a late summer or autumn occupation. Detailed analysis of the organic industry, the faunal remains and their stratigraphic position indicates that an older mammoth skeleton was present at the site on the arrival of the human occupants who used the tusks as raw material for implements^87^.

The flint at Maisières is dominantly black and of very high quality. It was sourced from local secondary deposits^89, 95, 101–103^. Knapping was geared toward producing large blades from bidirectional cores using soft stone hammers^96, 104^. These served as blanks for projectile points and various processing and craft tools, some of which were retouched and others used without secondary modification^84, 105–110^. A total of 945 retouched tools have been counted, among which burins (40.4%) and pointed tools (22.6%) dominate^96^ (Table S1). The 185 points can be further divided into three morphological categories: Maisières points (n = 121), tanged points (n = 56), and shouldered points (n = 8)^96^. Use-wear studies have shown that the points include both projectiles and knives^106, 108, 110^. Tanged points that are the focus here were shaped by a succession of direct removals that are sometimes very invasive^94–96, 104, 111^. Besides initial shaping of the limb, this retouch was also applied for the purposes of knife resharpening^110^. On projectile points, the investment in shaping highly depended on the original shape of the blank. Extensive retouch was applied when significant modification of morphology was necessary. The shaping technique enabled the precise adjustment of length, width, thickness, edge and tip angles, and the position of the tang^96^. In some cases, suitable distal shape was obtained through core preparation prior to blank detachment^104, 111^ and the limb of the point remained unretouched (Fig. S1).

The excellent preservation of the lithic points, their large number, their distinct morphologies, and recent technological analyses that allowed their careful experimental replication presented a unique opportunity to reconstruct the delivery system(s) with which the projectiles were shot. The recent faunal analyses^87^ provided the necessary background for designing the projectile experiments and for understanding links between weapon choice and hunting strategies.

## Results

In the absence of a reliable methodology for identifying weapon delivery systems based on impact fractures on stone points (see above and SI Text S1), our study protocol involved developing a new method and tailoring it to the Maisières-Canal archaeological sample (see Methods) to determine which weapon(s) were used by the Early Upper Paleolithic hunter-gatherers. The stepwise analytical protocol consisted of (1) identifying archaeological projectiles using strict criteria and recording impact fracture attributes and microwear evidence on them in order to reconstruct (2) the hafting system and (3) the weapon delivery system through experimental testing and verification. The results of each step are presented below. All the retouched points and their fragments, tanged burins, tang fragments, and an additional random sample of 100 unretouched blades, adding up to 329 artefacts, were screened for evidence of projectile use. Ninety-seven of the artefacts were selected for more detailed analysis, and a subsample were also studied for microwear.

1. *Projectile armatures and their impact fracture signature*

Among the 97 points subjected to the detailed analysis, a group of 17 tanged points, 11 Maisières points, and four distal fragments could be identified reliably as projectiles using strict criteria. This involved macroscopic and microscopic observation of patterns of impact breaks and scars and associated microwear features as well as the scoring of projectiles according to the frequency of attributes indicative of strongly compressive forces on breakage (see Methods). A total of 133 impact-related fractures were recorded on these artefacts. Because a morphologically homogeneous sample is required for weapon system reconstruction, we focus our attention here on 17 archaeological tanged points (14 near-intact points and three tang fragments).

**Figure 1:**
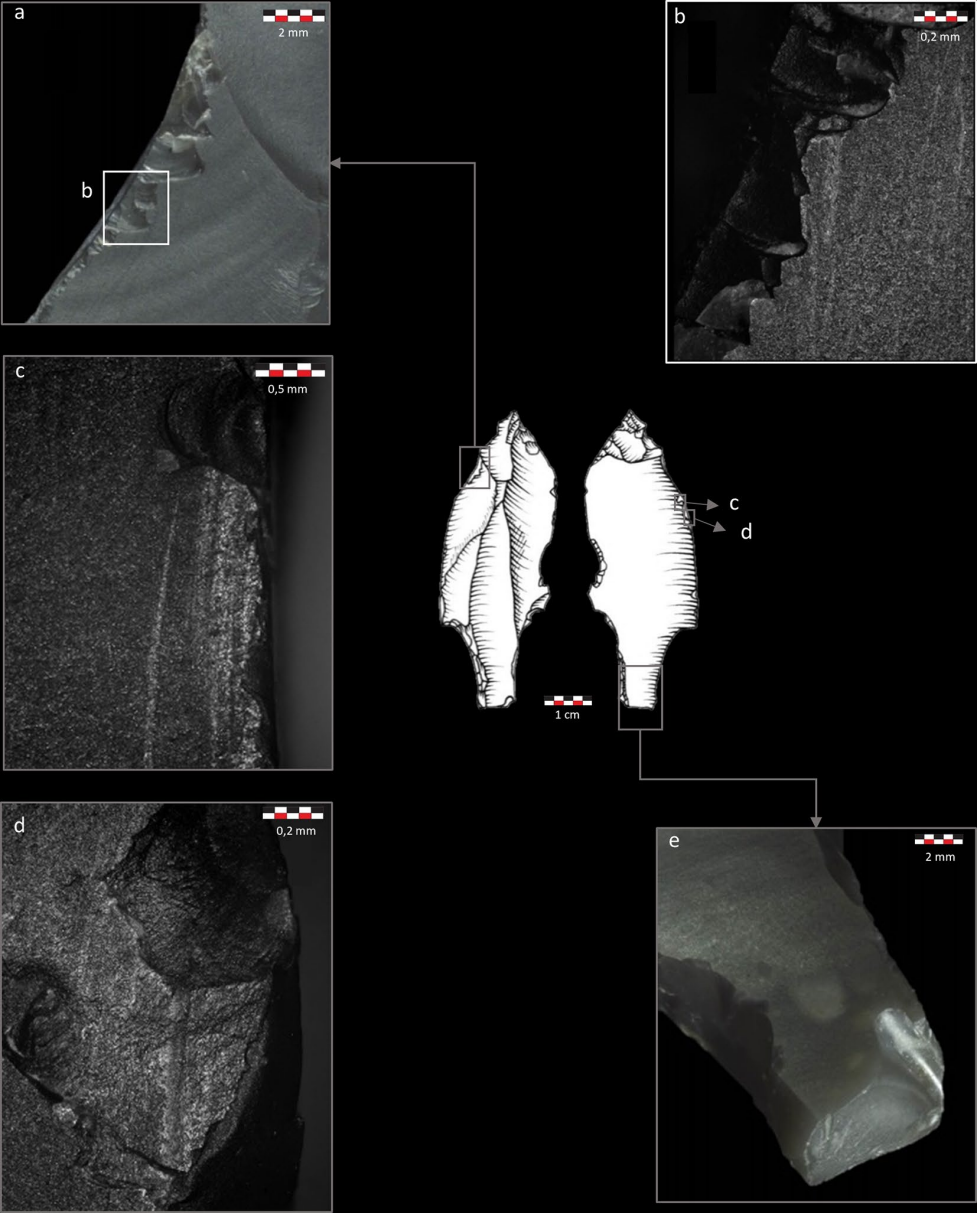
Tanged point P356.B6.1 identified as a projectile armature. (**a**) multiple diagonally oriented LS; (**b**) MLITs associated with edge damage shown in (**a**), oriented in the direction of scar propagation; (**c**,**d**) MLITs associated with LS on the limb; e: BB initiated on the ridge between the tang edge and the dorsal surface. Photos TraceoLab, drawing O. Touzé ^96^.

**Figure 2:**
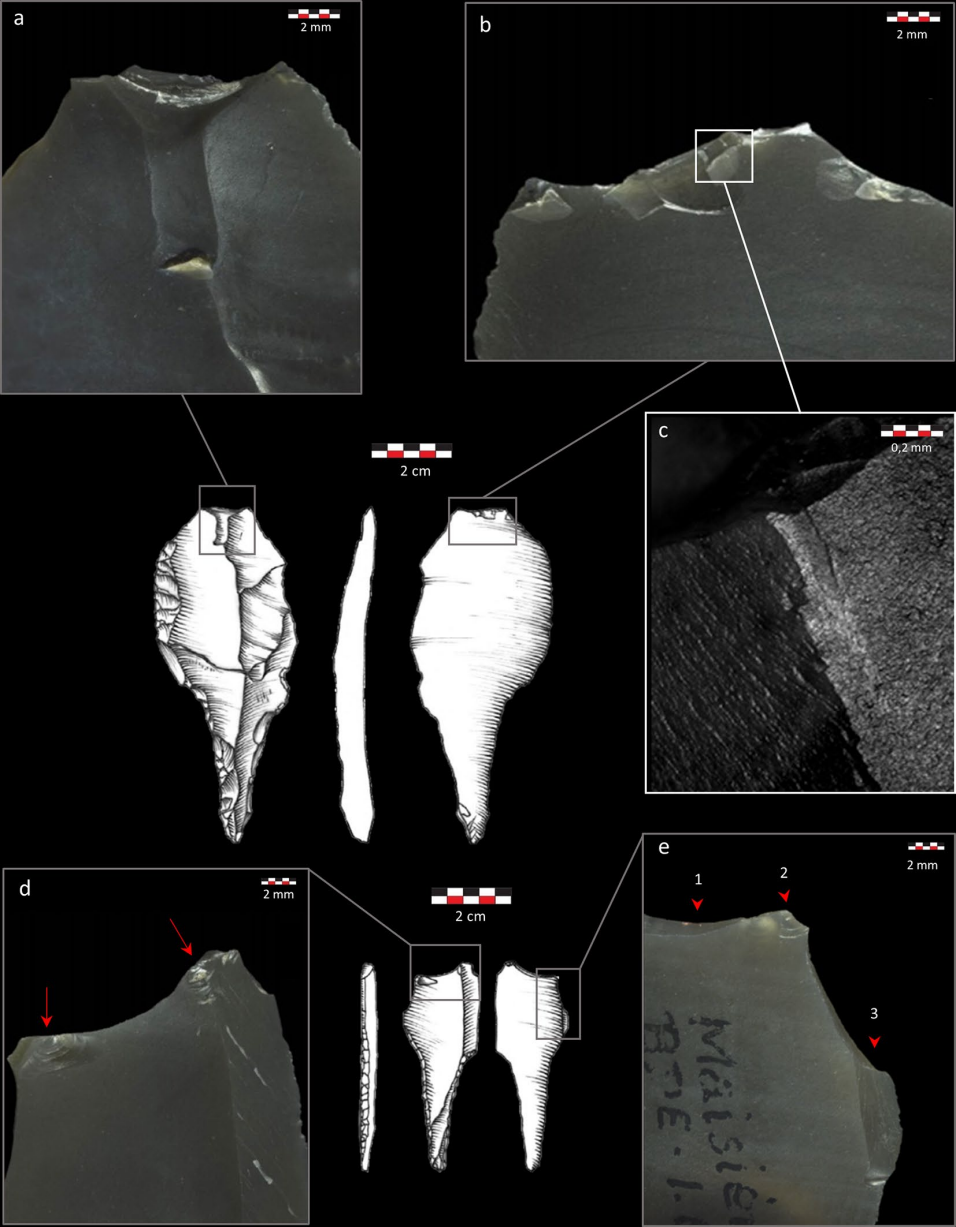
Tanged point P359.B2.2 identified as a projectile armature. (**a**) Ventrally initiated BB with a mixed initiation and a fissured hinge termination; (**b**) Multiple SD with mixed and bending initiations, initiated from the BB described in (**a**,**c**) MLITs associated with the multiple SD. Drawing by D. Pesesse ^104^. Bottom: tanged point P359.B2.13 identified as a projectile armature; (**d**) Several SD with cone initiations associated with the BB shown in (**e**); (**e**) Dorsally initiated BB with a snap termination (1); SD with cone initiations associated with the BB (2); indeterminate fracture (initiation removed) (3).

Sixty-six impact-related fractures were recorded on the 17 tanged points identified as definite projectiles (Table S2). Most fractures (48.5%) are lateral scars (LS) (Fig. 1a) that are in some cases associated with microscopic linear impact traces (MLITs)^112, 113^ (Fig. 1b–d). Other fracture features are bending breaks (BB) (Fig. 2a) (25.8%), secondary damage (SD) (Fig. 2b) (18.2%) that is sometimes associated with MLITs (Fig. 2c), and indeterminate fractures (Fig. 2e) (7.5%). Some of the BB are particularly noteworthy: four pieces (two tang fragments and two tanged points) present a BB initiated on the lateral side of the tang or on the ridge between the lateral side and the dorsal surface. The breaks terminate on the opposite edge and have a long propagation (Fig. 1e). They commonly have associated SD on one or several of the tang surfaces. The two pieces where the limb is preserved further show LS located on the distal edge of the limb and oriented diagonally toward the base (Fig. 1a). The tang breaks are highly informative of the stress conditions produced by the weapon on the moment of breakage. They testify to an impact stress applied perpendicularly to the axis of the tang, related to the stress applied to the distal edge of the limb for which the distal LS serve as evidence. This distinct fracture signal allowed proceeding to the reconstruction of the hafting system and the identification of the weapon delivery method.

2. *Hafting reconstruction*

The hafting system was hypothesized based on the morphological characteristics of the archaeological points and the results of the reactional impact stress (RIS) analysis, and then tested experimentally. Samples of experimental (n = 16) and archaeological points (n = 10) were also analyzed for hafting microwear (see Methods and SI Text S4.3).

The tanged points from Maisières-Canal are often made on curved blades^96^. Their tangs were shaped into a triangular or quadrangular cross-section. They have regular edges that taper toward the base and widen toward the limb. The curved profile of the points results in misalignment between the armature and the shaft if the point is hafted with dorsal/ventral shaft contact (Fig. S2a). These problems can be avoided by fitting the point in a split shaft so that the contact is with the lateral edges of the tang (Fig. S2b). Our RIS analysis found evidence on four tanged points of impact stress applied perpendicularly to the tang’s long axis. This type of stress can lead to breakage only if lateral movement of the tang is prevented by the shaft, i.e. when the shaft supports the lateral side(s) of the tang.

We tested this configuration in the first phase of experiments using three bonding techniques with different strengths: glue (mixture of 70% resin and 30% beeswax), sinew bindings, and their combination (Fig. S3), as the strength of the hafting system significantly influences fracture formation^113^. All the hafting solutions proved functional and allowed sufficient penetration into the target (Fig. S4). The proposed hafting configuration succeeded in producing a laterally initiated tang break associated with lateral scarring on the limb edge (Figs. 1 and S5) and thus confirmed that the archaeological fracture pattern is a consequence of lateral shaft contact. The tang break occurred only when the glue-sinew-glue (GSG) binding system was used. This implies that the archaeological hafting system was of a similar strength.

3. Weapon system identification

The tanged points from Maisières could hypothetically have functioned as components of any of the four traditional weapons: thrusting spear, throwing spear, spearthrower-and-dart, or bow-and-arrow. All four were tested experimentally. Bow could be ruled out first as an unlikely hypothesis on the grounds of the results of the shooting experiment and technical arguments related to the relationship between tang width and bow strength (Text S2 and Fig. S6). We consequently focused the latter part of the experimental program on the other three weapon systems. The experiments were carried out in three phases and were aimed at reproducing the fracture signal of the archaeological sample as closely as possible (see Methods).

Two main lines of evidence allowed identifying the spearthrower as the weapon responsible for the archaeological fracture signal. First, several characteristic tang breaks, always in association with lateral scarring on the limb, were produced experimentally and are consistent with the archaeological pattern. They only appeared when the projectile was shot with the spearthrower and made contact with resistant material (vertebra or scapula). There is a mechanical explanation as to why only the spearthrower produces this fracture combination. The weapon creates a relatively high angle of incidence, leading to reactional impact stress (RIS) with a pronounced bending component^114^. In addition, if the dart’s flight is abruptly halted by the target and its kinetic energy (KE) is not lost in the deformation or breakage of the target material, the fracturing of the stone tip, and/or the failure of the hafting system, the KE is dissipated in a bending movement of the shaft. This puts the tang under a significant bending stress. These combined stresses are responsible for the observed fracture pattern. The same phenomenon has been reported previously in experiments with Solutrean points (research program TFPS, *Technologie fonctionelle des pointes solutréennes*) and attributed to a lever effect produced by the long shaft^113^. This fracture pattern was recurrent in our archaeological and experimental samples, occurring on four out of 17 archaeological points (23.5%) and on five out of 20 experimental spearthrower darts (25%).

**Figure 3:**
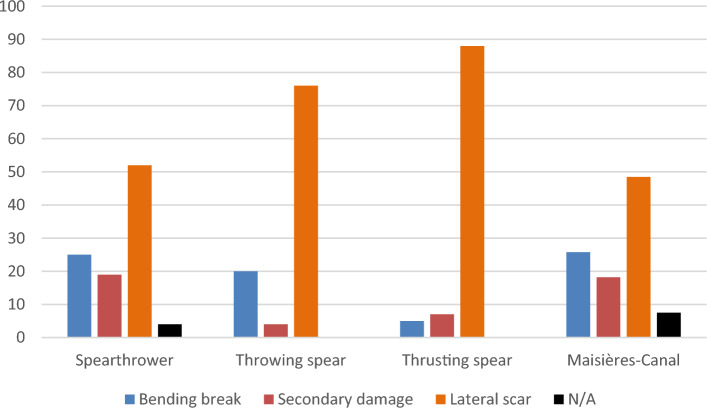
Relative fracture proportions (in %) for each weapon system from the two last phases of the experiments, contrasted with the fracture signal from Maisières-Canal (see Table S7 for the complete dataset).

The second line of evidence consists of the close resemblance between the relative frequencies of different fractures in the experimental spearthrower and the archaeological point sample and their dissimilarity to the experimental thrusting and throwing spear samples (Fig. 3). The close match with the spearthrower was achieved by adjusting experimental parameters through multiple steps to guarantee maximum comparability in point characteristics between the archaeological and experimental samples (see Methods). The similarity between the experimental spearthrower sample and the archaeological one was confirmed statistically (p = 0.951, χ^2^ = 0.100, df = 2), whereas the fracture signals produced by the thrusting spear and the throwing spear were significatively different from the archaeological sample (thrusting spear: p < 0.001, χ^2^ = 17.6, df = 2; throwing spear: p = 0.029, χ2 = 7.05, df = 2) (Tables S3–S6). Combined, the two lines of evidence make the spearthrower the best candidate for the weapon delivery system used at Maisières-Canal.

The slight difference between the experimental spearthrower sample and the archaeological projectile set—the higher percentage of LS in the experimental sample (Fig. 3, Table S7) —is best explained by details of weapon design. Our extended experimental dataset shows that in a morphologically homogeneous sample, the frequency of LS is influenced by the weight of the projectile used, with heavier projectiles showing more frequent LS^84^. Previous direct archaeological evidence and ethnographic data can give some guidelines for estimating the characteristics of the Maisières-Canal darts. Prehistoric darts and their fragments and complete spearthrowers have been recovered in Utah, Nevada, Arizona, New Mexico, Texas, and Arkansas^115^. The most complete examples come from White Dog Cave in Arizona. They consist of two intact spearthrowers and three darts, dated to around 2000 BP. The overall length of the darts is c. 140 cm^115^ and their reconstructed weight around 85 grams^116^, making them extremely light. The size and weight of ethnographic darts and spearthrowers vary significantly between contexts, but were generally adapted both to the prey and the body size of the hunter^117^. Most darts from the Arctic region weigh between 150 and 530 g and measure from 118 to 250 cm in length. Their Australian counterparts weigh between 250 and 624 g and are 190–460 cm long^117^. Our experimental darts weighed 160–300 g and were all 210 cm long (Table S8). The slight overrepresentation of LS on our experimental dart tips compared to the archaeological sample (Fig. 3, Table S7) could indicate that our experimental darts were somewhat heavier than the Early Upper Paleolithic models, which might approach the lighter end of the range documented for the Arctic.

## Discussion

Our study could establish the presence of spearthrower at Maisières-Canal around 28,000 BP/31,000 cal BP, which pushes back the dates for spearthrower use in Europe, as documented by direct evidence^42, 118^, by over 10,000 years. Representing the first attempt to carefully consider the idiosyncrasies of different prehistoric weapons in all their complexity, our results provide the thus far oldest reliable evidence for the use of long-range weaponry in prehistoric hunting.

In current debates on the emergence of long-range weaponry, the spearthrower has until recently^49, 119^ received relatively moderate attention compared to the bow even though both can be perceived as evolutionary gamechangers. These weapons have a similar range, and their flexibility in terms of prey choice and hunting strategies has been demonstrated ethnographically. Researchers have identified long-range weapons as potential contributors to increased diet breadth because they allow capturing small, fast game^22^, which has been perceived as an advantage when adapting to new environments^16^, or when coping with environmental stress or increased competition^120^.

In a recent synthesis grounded on the age of direct evidence of spearthrower use in Eurasia, Lombard hypothesizes the weapon as an LGM-driven innovation that was particularly adapted to hostile environments and selective range of prey and in such contexts may have had advantages over the bow^119^. Prior to the results we obtained on Maisières-Canal, numerous spearthrower hooks in osseous material were known from the Magdalenian in France and Spain, the oldest of them dated to around 20 cal BP. The hooks from Le Placard and Combe-Saunière are thought to be Solutrean^42, 118^, but their attribution is uncertain due to inconsistencies in the radiocarbon dates for Combe-Saunière level 1 and the excavation conditions at Le Placard^42^.

Our dataset places the spearthrower in a clearly pre-LGM context where, despite the relatively cold conditions, a rather wide range of fauna was exploited. The faunal remains from Maisières-Canal indicate an open landscape with a marshland component and semi-cold climatic conditions^87^. The large mammal remains include at least two reindeer, one horse, and one aurochs/bison. Small game is represented by a minimum of eight mountain hares and 17 birds^87, 99^.

The dominantly large size of the tanged points we analyzed allows associating them with hunting large rather than small game. The site has, nevertheless, also yielded pointed osseous artefacts, some of them tentatively interpreted as projectile armatures^87, 89, 111, 121^. If their function could be confirmed, we should consider the possibility that different dart designs, adapted to different kinds of prey, were employed by hunters at Maisières-Canal. Hunting by trapping has been previously proposed as an explanation for the frequency of small fauna at Maisières-Canal^87, 100^. The spearthrower may have offered a complementary strategy. Such flexibility would agree with the ethnographic record that depicts the spearthrower as a versatile weapon used mainly in open, yet varied, environments^25^. The Unalit Inuit in Alaska employed the weapon to hunt a range of prey from large marine mammals (e.g. seals, beluga whales) to marine birds^117, 122^. In Australia, it was used for hunting kangaroos, wallabies, emus, cranes, and ducks, and for fishing^117, 123^.

In addition to diversity in targeted prey, the ethnographic record also reflects variability in the social organization of spearthrower hunting. Individual ambush hunting and approach hunting employing blinds, as well as collective hunts where a small group of shooters were aided by several beaters who drove the game into a desired direction, have been reported^117, 123^. The flexibility of the weapon appears remarkable particularly when we consider that the ethnographic record is biased towards the Arctic and other environments that were hostile to agriculture and therefore allowed hunter-gatherers to persist up until historical times.

Put together, the ethnographic data and the archaeological context of the Maisières-Canal dart tips raise the question whether the spearthrower was a cold-adapted, specialized weapon responding to harshening environmental conditions in certain parts of the world towards the Last Glacial Maximum^119^ or whether its preferential occurrence in glacial contexts might rather reflect preservation and research bias. The use of the weapon at Maisières-Canal at 31 kya could either imply that it was at that point in time a relatively novel or rare weapon that gained in popularity as the climate turned colder and the landscape more open in the proximity of expanding glaciers in Europe. Alternatively, it could represent an innovation of a much greater antiquity that could adapt to extremely varied hunting situations and that is simply better visible in glacial contexts due to the better organic preservation towards the late Upper Palaeolithic and the spectacular, decorated spearthrower fragments known from this period.

Decades of anthropological analysis of living hunters-gatherers has documented an impressive variability in subsistence strategies^28^ and archaeologists increasingly acknowledge that hunters’ choice of weapon system depends not only on their technical competence and the available prey but also on socio-economic dynamics, and that different weapon systems can be used in combination^119^. Whether the Maisières-Canal assemblage represents an early or late example of spearthrower use by Paleolithic hunter-gatherers and to which ecological and social conditions it responded remains to be investigated by compiling comparable datasets for both older and younger periods.

Methods focused on lithic projectile armatures are the most adaptable in providing such data, as they are the least affected by preservation bias. Stone armatures, when studied with appropriate methods, hold similar potential than a bullet in a forensic investigation. They are brittle, so their breakage registers the stress conditions produced by different weapons. They are also durable, occurring across the entire Stone Age globally, and therefore provide an extensive record of past hunting behavior.

**Figure 4:**
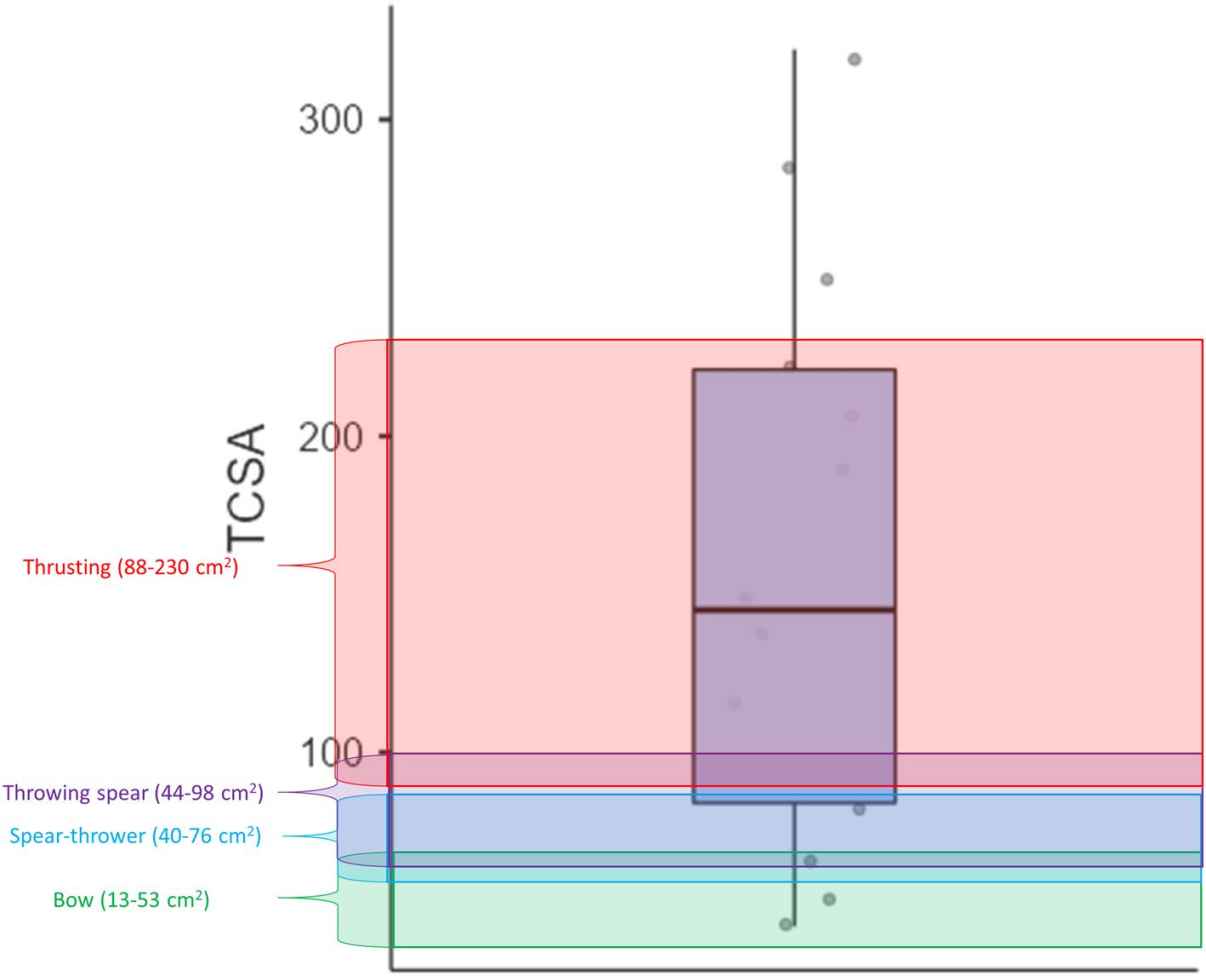
TCSA values in cm^2^ for the archaeological tanged points identified as spearthrower tips. The TCSA ranges of different weapon delivery systems are extracted from ^67^. Data for the boxplot is available in Table S9.

Despite this potential, recent claims for early appearance of long-range weaponry tend to heavily rely on arguments based on point size^36, 45, 48, 83^. The usefulness of the size argument is severely undermined by ethnographic datasets that show that size variability in spearthrower darts is so extensive that a large proportion of archaeological armatures deriving from different periods fit in it^77, 124^. The metrics of the spearthrower dart tips identified at Maisières-Canal conform to this pattern. Their TCSA values range from those proposed for the bow to those reported for the thrusting spear (Fig. 4, Table S9). This sheds further critical light on the size-based model for the development of early weaponry and implies that the emergence and co-existence of different weapon systems remains a vastly open question in prehistoric archaeology.

Here, we have presented an updated method that is based on trace evidence, builds on earlier experimental advances^75, 77–82, 114, 125–129^, is widely applicable, and can help decipher the archaeological record. When certain conditions can be fulfilled, i.e., when faunal data is available to reconstruct plausible targets for experimentation, when the sample of reliably identified archaeological armatures is sufficiently large (n > 15), and when armature raw material and morphology can be replicated, this method permits the identification of weapon delivery systems.

We contend that the current size-based view of the global variability of Stone Age hunting weaponry should be given the status of testable hypotheses^16^ and that weapon delivery system identification should rely on advanced, experiment-based analysis of direct traces of use on stone armatures because trace evidence carries more weight than analogical evidence (i.e. the popular size arguments). The future application of the method we have presented here has the potential to transform our view of the evolution of prehistoric weaponry. We have demonstrated here the power of the method by bringing forth the oldest reliable trace-based evidence for the use of spearthrower in the archaeological record.

## Materials and methods

### Archaeological sample

We studied a sample of 329 flint artefacts for evidence of weapon use. This included all the points (tanged points, Maisières points, backed pieces and fragments) in the collection, as well as tang fragments, a selection of tanged burins, and a random sample of 100 blades deriving from the main find concentration excavated at Maisières-Canal. The final sample for detailed macrofacture analysis was narrowed down to 97. A total of 32 pieces, including 14 tanged points, three tang fragments, 11 Maisières points, and four distal fragments, could be securely identified as projectile armatures (Table S10).

A new method to identify the weapon delivery system was designed and consisted of the following steps (see SI Text S4–5 for further detail):

1. **Screening of the archaeological collection and selection of a morphologically homogeneous group of potential armatures**. As projectile morphology is the most influential parameter in impact fracture formation (SI Text S3), the final sampling here targeted the tanged points from Maisières-Canal.
2. **Identification of pieces used as projectile elements**. Our projectile identification relied on observing sets of traces that combine into a wear pattern^84, 113^ using both low and high magnification observation (Text S4.1). Particularly fracture attributes indicative of compressive forces in armature breakage were sought for. A wear pattern with several compressive characteristics indicates that the pattern was created by impact. We used a previously developed scoring system that highlights compression features and establishes a threshold for reliable projectile identification (Text S4.2)^84^. This involved a complete recording of all the attributes for each fracture identified on the pieces (see Table S11 and Supplementary Information 2). A complementary high magnification analysis was carried out on 15 of the armatures to detect MLITs (microscopic linear impact traces), and 10 artefacts were also examined for hafting-related microwear (Text S4.3).
3. **Detailed morphometric and technological analysis of the securely identified archaeological projectiles**. Experiments are necessary to reconstruct the hafting system and delivery method of archaeological armatures, and the experimental pieces must replicate the morphometrics and technology of the archaeological pieces as closely as possible to guarantee the comparability of fracture signals^130^. We took advantage of the available technological and metric data^95, 96^ and carried out additional measurements when necessary to aid the experimental replication (Fig. S7).
4. **RIS (reactional impact stress) analysis**. Terminal ballistics concerns all the interacting phenomena that occur between the projectile and its target. Our weapon delivery system identification relied on the principle that different weapons create different reactional impact stress (RIS) conditions. We define RIS as the stress received by a projectile armature on impact following Newton’s third law of motion^114^. The RIS conditions affect fracture attributes in a systematic manner. The main challenge for RIS analysis is that the weapon system is not the most influential variable affecting fracture formation. A link can be established between a weapon system and a fracture signal only on the condition that the effects of other, more influential, parameters are controlled for.
  Previous works and our projectile experiments^84, 131, 132^ could establish that the parameters are, in order of importance, armature morphology, lithic raw material, hafting system, and weapon delivery system (Text S3). Because of this, the RIS analysis was carried out on a sample that was homogeneous in armature morphology and raw material (the tanged points). The hafting system was assumed to be constant, which was confirmed by a good match between the archaeological and experimental fracture patterns in the first experiment.
  The RIS analysis was based on a comparison of relative frequencies of three fracture families (Text S4.4). These were constructed from the attribute data, focusing on the following variables: *fracture amplitude, morphology of initiation, location of initiation, location of termination*, and *general direction of propagation*. Fractures can be divided into three main groups: bending breaks, secondary damage, and lateral scars^84^. The relative proportions of these have been found the most useful for distinguishing between weapon delivery systems after intensive analysis of data collected on the TRAIL projectile reference collection^131^, more specifically on a subset of 450 projectiles, 270 of which presented a total of 881 fractures.
  The category of bending breaks (BB) groups together all breaks (*fracture amplitude*) initiated in bending (*morphology of initiation*) on a surface or an edge (*location of initiation*) and terminating on the opposite surface or the edge (*location of termination*) regardless of their type of termination (Figs. 1e, 2a,e).
  Secondary damage (SD) is composed of scars (*fracture amplitude*). They initiate on a pre-existing bending break surface or on the abrupt termination of a pre-existing fracture (*location of initiation*) and terminate on a surface or an edge (*location of termination*). All such scars are included in this class regardless of their termination. Their orientation is the same as that of the original bending fracture (*general direction of propagation*) (Fig. 2b,d,e).
  Lateral scars (LS) include all scars (*fracture amplitude*) initiated on a surface (*location of initiation*) and terminated on the opposite surface (*location of termination*) that exhibit a common oblique or perpendicular orientation to the axis of the point (*general direction of propagation*). Scars with all kinds of initiations and terminations are included (Fig. 1a).
  This tri-partite classification yielded a fracture signal that was then reproduced experimentally to infer the weapon delivery system. Sample size is important in RIS analysis. Experimental studies have shown that impact-damaged armatures exhibit an average of two or three fractures depending on how fragile they are^84^. Minimum sample size is therefore set to 15 artefacts to obtain at least 30 fractures, which can be considered a minimum for the following steps of the analysis. Our sample of 17 tanged points fulfilled this criterion.
5. **Weapon design reconstruction**
  Reconstructing Paleolithic weapon design compares to solving an equation with two unknowns: the hafting system and the delivery system. In sample selection and experimental replication, point raw material and morphology were therefore held constant by choosing a raw material with highly similar fracture mechanical qualities and by replicating point morphology as closely as possible (Text S5.1). The two unknowns were then addressed by means of a sequential experimental program (Text S5). The first phase of experiments was aimed at determining the hafting system (Text S5.2) and the second (Text S5.3) and third phase (Text S5.4) were dedicated to reconstructing the weapon delivery method. The order of these phases was crucial because hafting system is the more influential of the two parameters in fracture formation, as it acts as a filter to the stress produced by the delivery system.
  A. *Hafting system*
    A hypothesis for hafting system was formulated based on the evidence (morphology of the pieces, fracture patterns, possible hafting traces) obtained during the morphometric and use-wear analysis of the archaeological material. Armature morphology (profile curvature) helped rule out improbable hafting configurations (Fig. S2). To test the hypothesis, an experiment was conducted that involved all possible delivery systems (the bow, the spear-thrower, the throwing spear, and the thrusting spear). The results were compared with the archaeological data using contingency tables on the relative proportions of the three facture categories defined above. The hafting system that produced a fracture signal closest to the archaeological sample was selected for the next steps of experimentation.
  B. *Delivery system*
    Hypothesis for the weapon delivery system was informed by the RIS analysis of the archaeological sample, previous experimental datasets on the TRAIL reference collection^84, 131^, and the results of the first phase of experiments. The subsequent steps were carried out to adjust the experimental parameters following the discrepancies observed between the archaeological sample and the first set of experimental projectiles. Here, a satisfactory match (documented by χ^2^ test) between the experimental spearthrower sample and the archaeological data could be achieved after increasing control over point morphology and raw material characteristics, and after ensuring that the match was not affected by a metric bias in the experimental sample (Text S5.3; S5.4).

### Supplementary Information

Supplementary Information 1.

Supplementary Information 2.

## Data Availability

All data generated or analyzed during this study are included in the Supplementary Information files except the raw description of the archaeological and experimental fractures. These datasets are available from the corresponding author on reasonable request.

## References

[1] Oakley KP, Andrews P, Keeley LH, Clark JD (1977). A reappraisal of the clacton spearpoint. Proc. Prehist. Soc..

[2] Milks A (2020). A review of ethnographic use of wooden spears and implications for Pleistocene hominin hunting. Open Quaternary.

[3] Thieme H (1997). Lower Palaeolithic hunting spears from Germany. Nature.

[4] Schoch WH, Bigga G, Böhner U, Richter P, Terberger T (2015). New insights on the wooden weapons from the Paleolithic site of Schöningen. J. Hum. Evol..

[5] Conard NJ (2015). Excavations at Schöningen and paradigm shifts in human evolution. J. Hum. Evol..

[6] Thieme H, Veil S, Meyer W, Möller J, Plisson H (1985). Neue Untersuchungen zum eemzeitlichen Elefanten–Jagdplatz Lehringen. Ldkr. Verden. Die Kd..

[7] Gaudzinski-Windheuser S (2018). Evidence for close-range hunting by last interglacial Neanderthals. Nat. Ecol. Evol..

[8] Rhodes JA, Churchill SE (2009). Throwing in the Middle and Upper Paleolithic: Inferences from an analysis of humeral retroversion. J. Hum. Evol..

[9] Rots V (2009). The functional analysis of the Mousterian and Micoquian assemblages of Sesselfelsgrotte, Germany: aspects of tool use and hafting in the European Late Middle Palaeolithic. Quartär.

[10] Villa P, Roebroeks W (2014). Neandertal demise: An archaeological analysis of the modern human superiority complex. PLoS One.

[11] Villa P, Soriano S (2010). Hunting weapons of Neanderthals and early modern humans in South Africa: Similarities and differences. J. Anthropol. Res..

[12] Sahle Y (2013). Earliest stone-tipped projectiles from the Ethiopian rift date to >279,000 years ago. PLoS One.

[13] Rots V (2013). Insights into early Middle Palaeolithic tool use and hafting in Western Europe. The functional analysis of level IIa of the early Middle Palaeolithic site of Biache-Saint-Vaast (France). J. Archaeol. Sci..

[14] Rots V, Coppe J, Conard NJ (2021). A Leaf Point Documents Hunting with Spears in the Middle Paleolithic at Hohle Fels, Germany. Mitteilungen der Gesellschaft für Urgeschichte.

[15] Soressi M, Locht J-L (2010). Les armes de chasse de Neandertal: Première analyse des pointes moustériennes d’Angé. Archéopages.

[16] Sisk ML, Shea JJ (2011). The African origin of complex projectile technology: An analysis using tip cross-sectional area and perimeter. Int. J. Evol. Biol..

[17] Shea JJ, Sisk ML (2010). Complex projectile technology and and homo sapiens dispersal into Western Eurasia. PaleoAnthropology.

[18] Shea JJ (1988). Spear points from the Middle Paleolithic of the Levant. J. F. Archaeol..

[19] Schmitt D, Churchill SE, Hylander WL (2003). Experimental evidence concerning spear use in Neandertals and early modern humans. J. Archaeol. Sci..

[20] Faivre JP (2014). Middle Pleistocene human remains from Tourville-la-Rivière (Normandy, France) and their archaeological context. PLoS One.

[21] Conard NJ, Serangeli J, Bigga G, Rots V (2020). A 300,000-year-old throwing stick from Schöningen, northern Germany, documents the evolution of human hunting. Nat. Ecol. Evol..

[22] Churchill SE (1993). Weapon technology, prey size selection, and hunting methods in modern hunter-gatherers: Implications for hunting in the palaeolithic and mesolithic. Archeol. Pap. Am. Anthropol. Assoc..

[23] Milks A, Champion S, Cowper E, Pope M, Carr D (2016). Early spears as thrusting weapons: Isolating force and impact velocities in human performance trials. J. Archaeol. Sci. Reports.

[24] Hitchcock, R. K. & Bleed, P. Each according to need and fashion: spear and arrow use among San hunters of the Kalahari. in *Projectile Technology* (ed. Knecht, H.) 345–368 (Plenum Press, 1997).

[25] Cattelain, P. Hunting during the Upper Paleolithic: bow, spearthrower, or both? in *Projectile Technology* (ed. Knecht, H.) 213–240 (Plenum Press, 1997).

[26] Nelson, M. C. Projectile points: form, function, and design. in *Projectile Technology* (ed. Knecht, H.) 371–384 (Plenum Press, 1997).

[27] Shott MJ (1993). Spears, darts, and arrows: Late woodland hunting techniques in the Upper Ohio Valley. Antiquity.

[28] Kelly, R. L. *The Lifeways of Hunter-Gatherers: The Foraging Spectrum* (Cambridge University Press, 2013).

[29] Knecht, H. The history and development of projectile technology research. in *Projectile Technology* (ed. Knecht, H.) 3–35 (Plenum Press, 1997).

[30] Marean CW, d’Erric F, Backwell LR, Tobias PV (2005). From the tropics to the colder climates: contrasting faunal exploitation adaptations of modern humans and Neanderthals. From Tools to Symbols: From Early Hominids to Modern Humans.

[31] O’Connell, J. F. How did modern humans displace Neanderthals? Insights from hunter-gatherer ethnography and archaeology. in *When Neanderthals and Modern Humans Met* (ed. Conard, N. J.) 43–64 (Kerns Verlag, 2006).

[32] Klein RG (2003). Whither the Neanderthals?. Science.

[33] Brown KS (2012). An early and enduring advanced technology originating 71,000 years ago in South Africa. Nature.

[34] McBrearty S, Brooks AS (2000). The revolution that wasn’t: A new interpretation of the origin of modern human behavior. J. Hum. Evol..

[35] Lombard M, Phillipson L (2010). Indications of bow and stone-tipped arrow use 64 000 years ago in KwaZulu-Natal, South Africa. Antiquity.

[36] Lombard M (2011). Quartz-tipped arrows older than 60 ka: Further use-trace evidence from Sibudu, KwaZulu-Natal, South Africa. J. Archaeol. Sci..

[37] Backwell L (2018). The antiquity of bow-and-arrow technology: Evidence from Middle Stone Age layers at Sibudu Cave. Antiquity.

[38] Bradfield J, Lombard M, Reynard J, Wurz S (2020). Further evidence for bow hunting and its implications more than 60 000 years ago: Results of a use-trace analysis of the bone point from Klasies River Main site, South Africa. Quat. Sci. Rev..

[39] Sahle Y, Brooks AS (2019). Assessment of complex projectiles in the early Late Pleistocene at Aduma, Ethiopia. PLoS One.

[40] Rust, A. *Die alt- und mittelsteinzeitlichen Funde von Stellmoor*. (Karl-Wachholtz Verlag, 1943).

[41] Meadows J, Heron C, Hüls M, Philippsen B, Weber M-J (2018). Dating the lost arrow shafts from Stellmoor. Quartär.

[42] Cattelain, P. The Le Placard spearthrowers. in *The Grotte du Placard at 150: New considerations on an exceptional prehistoric site* (ed. Delage, C.) 146–156 (Archaeopress, 2018).

[43] Cattelain, P. & Bellier, C. *La Chasse dans la Préhistoire du Paléolithique au Néolithique en Europe... et ailleurs (Nouvelle édition)*. (Éditions du CEDARC, 2002).

[44] Shea JJ (2006). The origins of lithic projectile point technology: Evidence from Africa, the Levant, and Europe. J. Archaeol. Sci..

[45] Metz, L., Lewis, J. E. & Slimak, L. Bow-and-arrow, technology of the first modern humans in Europe 54,000 years ago at Mandrin, France. *Sci. Adv.* **9**, eadd4675 (2023). 10.1126/sciadv.add4675 PMC9946345 36812314

[46] Shea, J. J. The impact of projectile weaponry on Late Pleistocene hominin evolution. in *The Evolution of Hominin Diets: Integrating Approaches to the Study of Palaeolithic Subsistence* (eds. Hublin, J.-J. & Richards, M. P.) 189–199 (Springer, 2009).

[47] Sano K (2016). Evidence for the use of the bow-and-arrow technology by the first modern humans in the Japanese islands. J. Archaeol. Sci. Rep..

[48] Sano K (2019). The earliest evidence for mechanically delivered projectile weapons in Europe. Nat. Ecol. Evol..

[49] Lombard M, Shea JJ (2021). Did Pleistocene Africans use the spearthrower-and-dart?. Evol. Anthropol..

[50] Rots V, Lentfer C, Schmid VC, Porraz G, Conard NJ (2017). Pressure flaking to serrate bifacial points for the hunt during the MIS5 at Sibudu Cave (South Africa). PLoS One.

[51] de la Peña P, Taipale N, Wadley L, Rots V (2018). A techno-functional perspective on quartz micro-notches in Sibudu’s Howiesons Poort reveals the use of barbs in hunting technology. J. Archaeol. Sci..

[52] Fernández-Marchena JL, Ollé A (2016). Microscopic analysis of technical and functional traces as a method for the use-wear analysis of rock crystal tools. Quat. Int..

[53] Taipale N, Rots V (2019). Breakage, scarring, scratches and explosions: understanding impact trace formation on quartz. Archaeol. Anthropol. Sci..

[54] Hughes SS (1998). Getting to the point: Evolutionary change in prehistoric weaponry. J. Archaeol. Method Theory.

[55] Riede F (2010). Hamburgian weapon delivery technology. Before Farming.

[56] Rios-Garaizar, J. Experimental and archeological observations of Northern Iberian Peninsula Middle Paleolithic Mousterian Point Assemblages. Testing the Potential Use of Throwing Spears Among Neanderthals. in *Multidisciplinary Approaches to the Study of Stone Age Weaponry* (eds. Iovita, R. & Sano, K.) 213–225 (Springer, 2016). doi:10.1007/978-94-017-7602-8_15.

[57] Wadley L (2008). The Howieson’s Poort industry of Sibudu Cave. South African Archaeol. Soc. Goodwin Ser..

[58] Villa, P. & Lenoir, M. Hunting and Hunting Weapons of the Lower and Middle Paleolithic of Europe. in *The Evolution of Hominin Diets: Integrating Approaches to the Study of Palaeolithic Subsistence* (eds. Hublin, J.-J. & Richards, M. P.) 59–85 (Springer, 2009).

[59] Villa P, Soressi M, Henshilwood CS, Mourre V (2009). The Still Bay points of Blombos Cave (South Africa). J. Archaeol. Sci..

[60] Wilkins J, Schoville BJ, Brown KS, Chazan M (2012). Evidence for early hafted hunting technology. Science.

[61] Villa P, Lenoir M (2006). Hunting weapons of the Middle Stone Age and the Middle Palaeolithic: spear points from Sibudu, Rose Cottage and Bouheben. South. African Humanit..

[62] Wadley L, Mohapi M (2008). A Segment is not a Monolith: Evidence from the Howiesons Poort of Sibudu, South Africa. J. Archaeol. Sci..

[63] de la Peña P, Wadley L, Lombard M (2013). Quartz Bifacial Points in the Howiesons Poort of Sibudu. South Afr. Archaeol. Bull..

[64] Lazuén T (2012). European Neanderthal stone hunting weapons reveal complex behaviour long before the appearance of modern humans. J. Archaeol. Sci..

[65] Hardy BL (2013). Impossible Neanderthals? Making string, throwing projectiles and catching small game during Marine Isotope Stage 4 (Abri du Maras, France). Quat. Sci. Rev..

[66] Lombard M (2020). Testing for poisoned arrows in the Middle Stone Age: A tip cross-sectional analysis of backed microliths from southern Africa. J. Archaeol. Sci. Reports.

[67] Lombard M (2021). Variation in hunting weaponry for more than 300,000 years: A tip cross-sectional area study of Middle Stone Age points from southern Africa. Quat. Sci. Rev..

[68] Lombard M, Pargeter J (2008). Hunting with Howiesons Poort segments: pilot experimental study and the functional interpretation of archaeological tools. J. Archaeol. Sci..

[69] Lombard M, Lotter MG, Caruana MV (2022). The Tip Cross-sectional Area (TCSA) method strengthened and constrained with ethno-historical material from Sub-Saharan Africa. J. Archaeol. Method Theory.

[70] Sahle Y, Morgan LE, Braun DR, Atnafu B, Hutchings WK (2014). Chronological and behavioral contexts of the earliest middle stone age in the gademotta formation, main ethiopian rift. Quat. Int..

[71] Douze K, Delagnes A, Wurz S, Henshilwood CS (2018). The Howiesons Poort lithic sequence of Klipdrift Shelter, southern Cape. South Africa. PLoS One.

[72] Douze K, Delagnes A, Rots V, Gravina B (2018). A reply to Sahle and Braun’s reply to ‘The pattern of emergence of a Middle Stone Age tradition at Gademotta and Kulkuletti (Ethiopia) through convergent tool and point technologies’ [J. Hum. Evol. 91 (2016) 93–121]. J. Hum. Evol..

[73] Hutchings, W. K. The Paleoindian fluted point: dart or spear armature? The identification of Paleoindian delivery technology through the analysis of lithic fracture velocity. (Simon Fraser University, 1997).

[74] Hutchings WK (2011). Measuring use-related fracture velocity in lithic armatures to identify spears, javelins, darts, and arrows. J. Archaeol. Sci..

[75] Iovita, R., Schönekeß, H., Gaudzinski-Windheuser, S. & Jäger, F. Identifying weapon delivery systems using macrofracture analysis and fracture propagation velocity: A controlled experiment. *Vertebr. Paleobiol. Paleoanthropology* 13–27 (2016). 10.1007/978-94-017-7602-8_2.

[76] Hutchings WK (2015). Finding the Paleoindian spearthrower: Quantitative evidence for mechanically-assisted propulsion of lithic armatures during the North American Paleoindian period. J. Archaeol. Sci..

[77] Clarkson, C. Testing Archaeological Approaches to Determining Past Projectile Delivery Systems Using Ethnographic and Experimental Data. in *Multidisciplinary Approaches to the Study of Stone Age Weaponry* (eds. Iovita, R. & Sano, K.) 189–201 (Springer, 2016).

[78] Sano, K. & Oba, M. Projectile experimentation for identifying hunting methods with replicas of Upper Palaeolithic weaponry from Japan. in *International Conference on Use-Wear Analysis: Use-Wear 2012* (eds. Marreiros, J., Bicho, N. & Gibaja, J. F.) 466–478 (Cambridge Scholars Publishing, 2014).

[79] Sano K, Oba M (2015). Backed point experiments for identifying mechanically-delivered armatures. J. Archaeol. Sci..

[80] Iovita R, Schönekess H, Gaudzinski-Windheuser S, Jäger F (2014). Projectile impact fractures and launching mechanisms: Results of a controlled ballistic experiment using replica Levallois points. J. Archaeol. Sci..

[81] Neill L, Clarkson C, Schoville B (2022). Holding your shape: Controlled tip fracture experiments on cast porcelain points. J. Archaeol. Sci. Rep..

[82] Sano, K., Denda, Y. & Oba, M. Experiments in Fracture Patterns and Impact Velocity with Replica Hunting Weapons from Japan. in *Multidisciplinary Approaches to the Study of Stone Age Weaponry* (eds. Iovita, R. & Sano, K.) 29–46 (Springer, 2016).

[83] Yaroshevich A, Kaufman D, Marks A (2021). Weapons in transition: Reappraisal of the origin of complex projectiles in the Levant based on the Boker Tachtit stratigraphic sequence. J. Archaeol. Sci..

[84] Coppe, J. Sur les traces de l’armement préhistorique : mise au point d’une méthode pour reconstruire les modes d’emmanchement et de propulsion des armatures lithiques par une approche expérimentale, mécanique et balistique. (Université de Liège, 2020).

[85] Haesaerts, P. & de Heinzelin, J. *Le site paléolithique de Maisières-Canal*. (De Tempel, 1979).

[86] Haesaerts P, Damblon F, Gerasimenko N, Spagna P, Pirson S (2016). The Late Pleistocene loess-palaeosol sequence of Middle Belgium. Quat. Int..

[87] Lacarrière, J. *et al.* A review of the Gravettian collections from the excavation of Maisières ‘Canal’ (Prov. of Hainaut, Belgium): A combined study of fossil and non-fossil animal resources for alimentary and technical exploitation. in *Les sociétés gravettiennes du Nord-Ouest européen : nouveaux sites, nouvelles données, nouvelles lectures - Gravettian societies in North-western Europe: new sites, new data, new readings. ERAUL 150* (eds. Touzé, O., Goutas, N., Salomon, H. & Noiret, P.) 23–51 (Presses Universitaires de Liège, 2021).

[88] Miller, R. Localisation et description du site de Maisières-Canal. in *L’atelier de taille aurigacien de Maisières-Canal (Belgique). ERAUL 110* (eds. Miller, R., Haesaerts, P. & Otte, M.) 7–12 (Études et Recherches Archéologiques de l’Université de Liège, 2004).

[89] de Heinzelin, J. *L’industrie du site paléolithique de Maisières-Canal*. (Institut royal des sciences naturelles de Belgique, 1973).

[90] Haesaerts, P. & Damblon, F. Les dates radiocarbone de Maisières-Canal. in *L’atelier de taille aurigacien de Maisières-Canal (Belgique). ERAUL 110* (eds. Miller, R., Haesaerts, P. & Otte, M.) 27–28 (Études et Recherches Archéologiques de l’Université de Liège, 2004).

[91] Jacobi RM, Higham TFG, Haesaerts P, Jadin I, Basell LS (2010). Radiocarbon chronology for the Early Gravettian of northern Europe: new AMS determinations for Maisières-Canal, Belgium. Antiquity.

[92] Lacarrière, J. Hit the north! Review of recent archaeozoological discoveries from Gravettian sites in the north of France. in *Les sociétés gravettiennes du Nord-Ouest européen : nouveaux sites, nouvelles données, nouvelles lectures - Gravettian societies in North-western Europe: new sites, new data, new readings. ERAUL 150* (eds. Touzé, O., Goutas, N., Salomon, H. & Noiret, P.) 53–73 (Presses Universitaires de Liège, 2021).

[93] Miller, R., Haesaerts, P. & Otte, M. *L’atelier de taille aurignacien de Maisières-Canal (Belgique). ERAUL 110*. (Études et Recherches Archéologiques de l’Université de Liège, 2004).

[94] Touzé O, Flas D, Pesesse D (2016). Technical diversity within the tanged-tool Gravettian: New results from Belgium. Quat. Int..

[95] Touzé, O. D’une tradition à l’autre, les débuts de la période gravettienne. Trajectoire technique des sociétés des chasseurs-cueilleurs d’Europe nord-occidentale. (Université de Liège/Université Paris 1 Panthéon-Sorbonne, 2019).

[96] Touzé O (2018). Aux prémices du Gravettien dans le Nord-Ouest européen: Étude de la production des pointes lithiques à Maisières- Canal (province de Hainaut, Belgique). Bull. la Société préhistorique française.

[97] Rasmussen SO (2014). A stratigraphic framework for abrupt climatic changes during the Last Glacial period based on three synchronized Greenland ice-core records: Refining and extending the INTIMATE event stratigraphy. Quat. Sci. Rev..

[98] Gautier, A. Mammifères fossiles. in *La faune du site paléolithique de Maisières-Canal* (eds. Gautier, A., Ballman, P. & de Coninck, J.) 3–20 (Institut royal des sciences naturelles de Belgique, 1973).

[99] Goffette Q, Lepers C, Jadin I, Rots V (2022). A handful of duck radiuses: Peculiarities of the avifaunal exploitation at the Gravettian site of Maisières-Canal (Belgium). Int. J. Osteoarchaeol..

[100] Gautier, A. Documentation paléontologique. in *Le site paléolithique de Maisières-Canal* (eds. Haesaerts, P. & de Heinzelin, J.) 66–68 (De Tempel, 1979).

[101] Miller, R. *Lithic Resource Management during the Belgian Early Upper Paleolithic. Effects of Variable Raw Material Context on Lithic Economy. ERAUL 91*. (Études et Recherches Archéologiques de l’Université de Liège, 2001).

[102] Moreau L, Hauzeur A, Jadin IL (2013). gestion des ressources lithiques dans l’ensemble gravettien de Maisières-Canal (Bassin de Mons, Hainaut, B) Nouvelles perspectives. Notae Praehistoricae.

[103] Moreau L (2016). Geochemical sourcing of flint artifacts from Western Belgium and the German rhineland: Testing hypotheses on gravettian period mobility and raw material economy. Geoarchaeology.

[104] Pesesse D, Flas D (2012). The Maisierian, at the edge of the Gravettian. Proc. Prehist. Soc..

[105] Michel, M. Entre altération et fonction : Impact des processus post-dépositionnels sur la conservation et la reconnaissance des traces d’utilisation des outils lithiques. Vers une meilleure compréhension fonctionnelle des sites gravettiens de l’ouest de l’Europe. (University of Liège, 2022).

[106] Rots, V. Hafting Traces on Flint Tools: Possibilities and Limitations of Macro- and Microscopic Approaches. (Katholieke Universiteit Leuven, 2002).

[107] Rots V (2002). Are tangs morphological adaptations in view of hafting? Macro- and microscopic wear analysis on a selection of tanged burins from Maisières-Canal. Notae Praehistoricae.

[108] Taipale, N. Hafting as a flexible strategy: variability in stone tool use and hafting at three European Upper Palaeolithic sites. (University of Liège, 2020).

[109] Taipale N, Rots V (2020). Revisiting Maisières-Canal (Hainaut, BE): New results on tool use and hafting. Notae Praehistoricae.

[110] Taipale N, Rots V (2021). Every hunter needs a knife: Hafted butchering knives from Maisières-Canal and their effect on lithic assemblage characteristics. J. Archaeol. Sci. Reports.

[111] Otte, M. Documentation archéologique. in *Le site paléolithique de Maisières-Canal* (eds. Haesaerts, P. & de Heinzelin, J.) (De Tempel, 1979).

[112] Moss, E. H. *The functional analysis of flint implements. Pincevent and Pont d’Ambon: two case studies from the French Final Palaeolithic. BAR International Series 177*. (Archaeopress, 1983).

[113] Rots V, Plisson H (2014). Projectiles and the abuse of the use-wear method in a search for impact. J. Archaeol. Sci..

[114] Coppe J, Lepers C, Rots V (2022). Projectiles Under a New Angle: A Ballistic Analysis Provides an Important Building Block to Grasp Paleolithic Weapon Technology. J. Archaeol. Method Theory.

[115] Pettigrew DB, Garnett J (2015). Atlatls and darts of white dog cave. Arizona. The Atlatl.

[116] Whittaker JC, Pettigrew DB, Grohsmeyer RJ (2017). Atlatl dart velocity: Accurate measurements and implications for paleoindian and archaic archaeology. PaleoAmerica.

[117] Cattelain PL (1994). chasse au Paléolithique supérieur: Arc ou propulseur, ou les deux ?. Archéo-Situla.

[118] Cattelain P (1989). Un crochet de propulseur solutréen de la grotte de Combe-Saunière 1 (Dordogne). Bull. la Société préhistorique française.

[119] Lombard M (2022). Re-considering the origins of Old World spearthrower-and-dart hunting. Quat. Sci. Rev..

[120] Henshilwood CS, Marean CW (2003). The origin of modern human behavior: Critique of the models and their test implications. Curr. Anthropol..

[121] Otte, M. *Le Paléolithique supérieur ancien en Belgique*. (Musées royaux d’art et d’histoire, 1979).

[122] Nelson EW (1902). The Eskimo about Bering Strait. Eighteenth annual report of the Bureau of American Ethnology.

[123] Cattelain P (2000). L’apport de la comparaison ethnographique à la connaissance et aux tentatives de reconstitution des propulseurs paléolithiques. Anthropol. Préhistoire.

[124] Newman K, Moore MW (2013). Ballistically anomalous stone projectile points in Australia. J. Archaeol. Sci..

[125] Coppe J, Rots V (2017). Focus on the target. The importance of a transparent fracture terminology for understanding projectile points and projecting modes. J. Archaeol. Sci. Rep..

[126] Coppe J (2019). Ballistic study tackles kinetic energy values of palaeolithic weaponry. Archaeometry.

[127] Pargeter J, Shea JJ, Utting B (2016). Quartz backed tools as arrowheads and hand-cast spearheads: Hunting experiments and macro-fracture analysis. J. Archaeol. Sci..

[128] Fischer A, Vemming Hansen P, Rasmussen P (1984). Macro and micro wear traces on lithic projectile points: Experimental results and prehistoric examples. J. Danish Archaeol..

[129] Soriano S (1998). Les microgravettes du Périgordien de Rabier à Lanquais (Dordogne): Analyse technologique fonctionnelle. Gall. Préhistoire.

[130] Geneste, J.-M. & Maury, S. Contributions of multidisciplinary experimentation to the study of Upper Paleolithic projectile points. in *Projectile Technology* (ed. Knecht, H.) (Plenum Press, 1997).

[131] Rots, V. TRAIL: An Experimental Trace and Residue Reference Library for the functional analysis of stone tools in Liège. *OSF Prepr.* 1–8 (2021). 10.31219/osf.io/jsak6.

[132] Tomasso S (2021). A closer look at an eroded dune landscape: first functional insights into the Federmessergruppen site of Lommel-Maatheide. Peer Community J..

