# Supplementary material for: Terminal ballistic analysis of impact fractures reveals the use of spearthrower 31 ky ago at Maisières-Canal, Belgium
Source: Sci Rep. 2023 Oct 25;13:18305. doi: 10.1038/s41598-023-45554-w (PMC10600151; doi:10.1038/s41598-023-45554-w)
Supplement: Supplementary file 1 — Supplementary Information 1. [file 41598_2023_45554_MOESM1_ESM.docx]

**Supporting Information for**

Terminal ballistic analysis of impact fractures reveals the use of spearthrower 31 ky ago at Maisières-Canal, Belgium

Justin Coppe *^a^, Noora Taipale ^a,b^, Veerle Rots ^a,b^

^a^ TraceoLab / Prehistory, University of Liège, Place du 20-Août 7 (Bât. A4), 4000 Liège, Belgium

^b^ F.R.S.-FNRS, Belgium

*Justin Coppe

Email: justin.coppe@uliege.be

Supporting Information Text S1-S5

**Text S1. Methodological aspects of prehistoric weapon system identification**

**S1.1 Projectile point size-based approach**

The fundamental assumption in using TCSA values for weapon system identification is that a projectile point must be larger than its shaft to enable effective penetration required of any weapon that causes fatal injury by blood loss. The maximum cross-sectional area (or perimeter) of a stone point has been considered a reliable predictor of shaft diameter, and shaft dimensions have been linked to weapon systems by way of comparison with ethnographic and/or experimental reference material^1^. Several critical reviews, however, have found that the distinct TCSA ranges proposed for different weapons are not realistic in the light of extended ethnographic datasets and probably underestimate prehistoric variability^2–4^, with the highly variable size of ethnographic spear thrower darts being particularly illustrative of this problem^2^.

Further, there are currently no grounds to assume that a projectile point must be wider than the maximum diameter of its shaft to function because reducing the shaft diameter toward the tip with the help of, for instance, a foreshaft is a way to haft small points on large shafts. Such is the case with the Roman pilum. The crucial aspect is to guarantee a smooth transition from point to shaft to avoid the so-called hilt effect^see 5^. Employing such adjustment of shaft design, we tested the effectiveness of small projectile tips (microgravettes) as thrusting spear and hand-cast spear tips, obtaining sufficient penetration to cause fatal injury on medium-sized and large game^6^. From this it follows that the assumption that a weapon tip must always be wider than its shaft to enable effective penetration is flawed. Consequently, point size alone cannot be considered diagnostic of weapon delivery systems.

**S1.2 Macrofractures**

Decades of experimental work have been devoted to investigating the relationship between projectile use and fracture formation on stone points^e.g. 7–16^. These studies were initially focused on establishing criteria for projectile identification but also laid the foundation for more advanced analysis geared toward, for instance, the identification of weapon delivery systems. More recently, numerous experiments have addressed the question of the relationship between weapon delivery systems and impact fracture characteristics^2,17–22^. These studies aimed to meticulously control the experimental setup, repeatedly employing crossbows^17,18,21,22^ or airguns^19^ to isolate the impact of velocity and kinetic energy on fracture formation. The authors examined their experimental projectiles for the frequency^17,18,22^ and relative proportions of different impact fractures^19,20^, their length^2,17,19–21,23^ or surface area^23,24^, their initiations and terminations^19,20^, the frequency of microscopic linear impact traces (MLITs)^18^, and relative loss of mass^18^. The results of these experiments are consistent and suggest a link between kinetic energy and both the frequency and length of fractures under controlled conditions^2,17–22^. Consequently, some researchers have inferred that fracture and MLIT frequency^17,18,22^ as well as fracture size^2,17–22^ on stone artifacts could serve as promising indicators of long-range weapons (bow and spearthrower).

Machine-assisted experiments are invaluable in elucidating the effects of individual parameters like kinetic energy on fracture formation. However, they tend to oversimplify the complex differences in the stress exerted on lithic projectiles by different weapons by reducing these differences to variation in velocity or kinetic energy. This has been acknowledged by several authors of the pioneering experimental studies, who in discussing their results draw the attention to multiple key variables influencing fracture formation, including point morphology, lithic raw material properties, contact with the target, hafting system, and impact angle^2,18,22^. The significance of point morphology^13,25^ is highlighted by authors who either knapped close replicas of their archaeological armatures^13,17,18,22^ or went one step further and eliminated the unwanted variation by employing near-identical casts of original archaeological points in glass^19^ or clay^21^. The strategy opted for in each case was achieving control over morphology rather than testing its influence. In contrast, the effect of impact angle has been studied in depth, with Iovita et al.^19^ reporting that the link between increased kinetic energy and longer fractures disappears when impact angle deviates from 90 degrees.

This warrants a scrutiny of the experimental and archaeological results obtained so far. Both crossbows and airguns produce a minimal angle of incidence, similar to the angle produced by a human archer shooting a longbow (angles between 1 and 3 degrees). Such a minimal angle of incidence results in impact stress that predominantly manifests as compression, leading to the dissipation of most of the kinetic energy along the axis of the point. This compression-dominated stress pattern is characteristic of bow-induced damage. In contrast, spearthrowers (with angles of incidence between 1 and 5 degrees) and especially throwing spears (angles ranging from 4 to 24 degrees) generate impact stress characterized by a combination of compressive and bending components. The degree to which kinetic energy is dissipated along the point's axis varies depending on the angle of incidence so that the higher the angle, the lesser the compressive component, and logically, the shorter the fracture propagation (see fig. 6 and 11 in^26^). Therefore, the ballistic differences between bows, spearthrowers, throwing spears, and thrusting spears are more intricate than mere variations in kinetic energy, necessitating a comprehensive understanding of their flight behavior^26,27^. This is best achieved by combining machine-assisted and actualistic experiments, the latter involving human shooters. Recent efforts have been initiated in this direction, with data beginning to emerge^2,21^. Notably, when human and machine-shot projectiles are contrasted, fractures terminating on the edges of the artifacts appear to hold greater potential for weapon diagnostics than other fracture categories^2^, and relative fracture length (fracture length divided by artifact length) appears to be a more promising indicator than raw fracture length^21^.

Another influential, yet underexplored, variable affecting impact fracture formation is the strength and design of the hafting system, as the kinetic energy available for the breakage of the lithic point depends on the quantity of energy lost in the deformation of the target and the possible partial or complete failure of the hafting system^25^. Large-scale projectile experiments carried out thus far have maintained the hafting system constant, often employing industrial raw materials, to mitigate the effect of variation introduced by this variable (see^28^). This is a necessary step in methodological investigations aimed at isolating and studying individual variables, such as kinetic energy. For the experimental insights to be archaeologically applicable, however, the researcher would need to demonstrate that the hafting system of prehistoric projectiles were likewise invariable and that the properties of the experimental and archaeological raw materials are a close enough match.

In sum, while significant progress has been made through multiple experimental programs in the recent years^2,17–22^, the experimental datasets that were available to the authors of recent studies focused on Early Upper Paleolithic weaponry^29–31^ derived from machine-assisted setups that model prehistoric projectile impact in a simplified manner. Therefore, while otherwise valuable, the data from these experiments is not directly usable for archaeological interpretation. It has not been demonstrated that the elevated frequency and magnitude of impact damage, cited as diagnostic of long-range weaponry by several authors^29–31^, are truly characteristic of the particular stress conditions generated by human-operated bows and spearthrowers. We believe they are simply reflective of the added kinetic energy in settings where other variables were held constant.

**S1.3 Fracture proportions**

The present study is not the first to investigate the link between relative fracture proportions and weapon delivery modes. Fischer et al.^7^ explored this topic, but their spear sample (11 Brommian points) was too small to discern a clear pattern. In a more recent study, Iovita and colleagues observed certain differences between low and high-velocity projectiles, but predicted these effects to diminish with modification of the experimental setup (e.g. a more realistic target)^19^. Sano and Oba^18^ presented results where backed points were shot with a crossbow at velocities typical of bows and spearthrowers, resulting in a proportionally higher occurrence of spin-offs (termed secondary damage in our study; for terminology, see^32^) compared to shots delivered by thrusting motion by a human experimenter or by the crossbow velocities replicating hand-cast spears. A similar outcome was later published where spin-offs were exclusively observed on transverse points fired with a crossbow at a velocity typical of bow^22^. Pargeter et al. also identified differences in fracture proportions produced by bows and throwing spears in their experimental study involving transverse quartz points^23^. However, their findings contradicted those of Sano and Oba^18,22^. They observed a relatively higher proportion of spin-offs in their throwing spear sample compared to their bow sample. They attributed this disparity to the differences in point raw material and morphology^23^.

An overarching evaluation of the results of these previous studies is complicated by the authors’ choice of terminology, for instance the use of fracture categories such as “burination” or “flute-like fracture”, which are uninformative of fracture attributes (e.g. location and type of initiation, primary vs secondary fracture, surface of termination) that would be relevant for reconstructing the mechanical stress conditions the lithic armature was subjected to. This issue is discussed in length elsewhere^32^.

More importantly, as far as we are aware, a comparison between experimental and archaeological fracture proportions has been attempted only twice (Table I and II in^33^ and supplementary Table 2 in^30^) and did not produce a match between the archaeological and the experimental data in either case. Soriano’s^33^ experimental points were shot exclusively with a bow, which might explain the discrepancy between his experimental and archaeological fracture proportions. In Sano’s case^30^, kinetic energy of the projectiles was altered to simulate different weapon systems, but the launching mechanism remained the same apart from the human thrusting experiment, for which little detail is provided in publications^17,18,22^. Their choice of launching mechanism does not replicate the actual variation between prehistoric weapons, which is primarily due to the angle of incidence^26^. In their archaeological case study, Sano and colleagues compared the experimental and archaeological datasets, but his identification of weapon delivery systems is based on arguments other than fracture proportions^30^. Consequently, no attempt has yet been successful in employing relative fracture frequencies to identify prehistoric weapon delivery modes.

The difficulties encountered by previous investigators stem from the fact that the delivery method is not the sole parameter influencing impact fracture formation; its effect is, in fact, the most subtle of all (Text S3). Therefore, the challenge lies in disentangling the stress generated by the delivery method from the more dominant influences of point morphology, raw material, and hafting system. This can be achieved if the analysis is backed up with a sequential experimental program. Such an experiment must replicate closely the archaeological point morphology and raw material and first reconstruct the hafting system through experimental tests. The influence of the weapon system on fracture patterns can be expected to become visible only when the more influential parameters are controlled for. The aim of our study is to further develop this approach through an archaeological case study and targeted experimental program.

**Text S2. Weapon system identification: ruling out the bow**

Our argument to exclude the bow from our analysis is primarily based on the technical limitations associated with arrow shafts. The size of the archaeological tangs from Maisières-Canal sets a lower limit to the dimensions of the shafts on which they could have been feasibly hafted. Their widths range from 7 to 18 millimeters, with a median width around 10 millimeters (Fig. S6). To accommodate the larger tangs (11-18 mm), represented by 6 pieces out of 12 that allowed measurement, with a side-contact hafting system, we would need to imagine arrow shafts with a diameter of approximately 10-15 mm.

We cannot rule out the possibility that Paleolithic hunters used such thick arrow shafts, but we consider it to be highly unlikely. While archaeological evidence does show arrow shafts with these diameters, they are typically associated with heavy warbows from the 16th century, as arrow shaft diameter corresponds to the draw weight of the bow to guarantee an acceptable spine value^26,34^. These bows had draw weights ranging from 110 to 186 pounds and a 30-inch draw length (p. 627, Table 8.26 in^35^). They are among the most powerful bows ever discovered and are known for shooting heavy arrows (around 100 g), generating a significant amount of kinetic energy, typically between 110 and 130 joules^36^. These bows are associated with Late Medieval warfare.

The existence of such strong bows in the Paleolithic seems unlikely. Most Neolithic bows have an estimated draw weight between 24 and 84 lb^37^. This range sets the maximum shaft diameter to between 6 and 10 mm. In the set of the 12 arrows we produced for the Maisières-Canal experiment, we never succeeded in hafting a tang wider than 10 mm on a shaft corresponding to these dimensions, which confirms the technical limit and makes arrows an unlikely hypothesis considering that only a small portion of the archaeological tangs falls below the 10 mm limit.

In addition, arrowheads used with the warbows from the 16th century were consistently equipped with sockets into which the arrow shafts were placed. At this energy level, a split shaft with an inserted tang (the hafting arrangement we propose for Maisières-Canal based on wear analysis and experimental verification) would not function effectively, as the shaft would frequently split upon impact due to the tremendous kinetic energy.

Nevertheless, even though we regarded the bow as an unlikely hypothesis for the technical reasons outlined above, we conducted tests on a sample of arrows (n=12). Our aim was to determine whether the distinct fracture pattern we observed experimentally for the spear-thrower (characterized by edge-to-edge tang breaks with secondary damage, consistently associated with lateral scars on the limb) could also manifest in the case of bow. This pattern did not occur, which further justified concentrating our efforts on the three other delivery systems for the remaining part of the experimental program.

**Text S3. TRAIL projectile collection**

Previous works on fracture mechanics of brittle solids have demonstrated the importance of the morphology of the surface beneath which the fracture front propagates for fracture attributes^38–40^. The fracture mechanical properties of the raw material are another essential variable^40,41^, also documented by our recent fracture tests^42^.

Our large-scale projectile experiments included in TRAIL^43^ evaluated the order of importance of these and other central parameters by testing their influence on fracture formation on a total of 560 projectiles produced in a single raw material. Three different point morphologies (triangular, bifacial, backed point) in two size categories were included, as well as three different hafting systems. These points were shot using all four modes of propulsion (thrusting, throwing, spearthrower, bow). A detailed study of all wear traces produced on this set of points confirmed the primary influence of point morphology, followed by lithic raw material, then the hafting system that modifies the stress the armature receives according to its strength and configuration (orientation and position of armature on the shaft), leaving the weapon delivery system as the least influential parameter^6,44^.

**Text S4 Methods**

**Text S4.1 Analytical technique and equipment**

The archaeological artefacts were analyzed under low magnification at the Royal Belgian institute of Natural Sciences in Brussels using two stereomicroscopes, a Leica S9i (magnifications from 6.1× to 50×) and a Nikon 229030 (magnifications 8–40×) with oblique lighting. The experimental points were studied at TraceoLab using a stereomicroscope Zeiss V12 (8-100×) and features photographed with a Zeiss V16 macroscope (5.6-180×). All the observed fractures were described in detail following the attribute-based terminology^6,32^. The high magnification analysis of the archaeological artefacts was carried out at the RBINS with an Olympus BH2-UMA metallurgical incident light microscope (magnifications 50–500×). The experimental pieces were studied and the microscopic photos taken at TraceoLab using a Zeiss AxioImager microscope and AxioVision software at 50× and 100× magnification^45^.

**Text S4.2 Summary of the scoring system for projectile identification**

The artefact must present a combination of at least two fractures to be considered for this analysis. Points with a single fracture, regardless of fracture characteristics, are not considered^25^. Fracture data serving as the basis of the scoring is collected using attribute recording outlined previously^32^. To increase the objectivity of projectile identification, we developed a scoring system^6^ that gives each artefact points according to the degree to which damage on it evidences compressive forces on breakage. We summarize the system here.

Each bending break (BB) yields one point if they present a feather, hinge, step, or complex termination. Snap-terminated BB do not increase the score because snap fractures lack a propagation subparallel to the termination surface. Consequently, they do not imply compressive stress.

Each BB that shows a termination other than snap receives an extra point if:

- *The break’s propagation is longer than the thickness of its cross-section* (a long propagation on a thin piece indicates a strong compressive component in the stress that produced the fracture).
- *Its abrupt termination (step, hinge, and complex) is fissured*. This occurs when, toward the end of its propagation, the crack retains enough compressive energy to propagate slightly further into the material before the bending component takes over and changes the direction of the fracture path, resulting in a step or hinge termination. This phenomenon is likewise indicative of a significant compressive component in the fracture stress.

Secondary damage (SD) is considered significant and earns a point if the scars present an orientation that is consistent with the other fractures on the piece (the direction is irrelevant in this case). One additional point is granted to SD for each of the indicators below:

- *The SD presents a fissured termination* (this is significant for the reasons outlined above);
- *The length of their propagation is longer than the thickness of the cross-section of the break from which they initiate*. SD can be created under pure bending stress^7^ if the piece is sufficiently thick to produce compressive stress on the concave surface during bending. According to the observations made on the TRAIL reference set, SD generated by pure bending stress rarely show propagations longer than the thickness of the primary break’s cross-section. Furthermore, SD with a long propagation can only be created on a thin piece when strong compressive stress is involved in the breakage.
- *There are multiple SD*. Several scars are initiated by prolonged contact between the two fracture surfaces (the positive and the negative part) under intense compressive stress.
- *The SD lack initiations because these were erased by subsequent scars*. A removed initiation is an indication of prolonged contact between the positive and negative fragments. Prolonged contact is favored by compressive stress because the two fragments remain aligned during the secondary fracture process.
- *The SD are located on the same surface from which the BB they are associated with initiates*. SD can be created by pure bending stress (see above), but under these conditions, they exclusively form on the termination surface of the BB from which they start. Since the positive and negative surfaces of the break move away from each other on the initiation side, they do not come into contact under a pure bending stress. Consequently, SD that form on the initiation surface can only be caused by contact between the two fracture surfaces, after the initial bending break. They therefore signal a notable compressive component in the stress that produced the fracture.
- *The SD are initiated on the abrupt termination of an earlier fracture*. This situation is observed when the distal extremity of the fracture positive strongly compresses the termination of its negative upon detachment. This phenomenon suggests a fracture stress with a great compressive component.

Lateral scars (LS), just like SD, earn a point if the direction of their propagation corresponds to the other fractures on the artifact. Lateral scars can have many causes but only a limited amount of those can lead to every scar laying in the same direction. One of these causes is impact.

Each of the LS occurrences receives a supplementary point if:

- *Multiple LS are visible*.
- *Their initiations are absent*.
- *They present fissured terminations*.
- *They present a propagation that is longer than the thickness of their cross-section*.

The mechanical justifications for these criteria are the same as those given for SE and BB above.

The scoring allows to weigh the relative importance of each attribute and to evaluate to what extent the fractures are representative of compressive forces. The final score reflects the certainty with which the fractures can be attributed to projectile impact. A sum between 0 and 3 points is insufficient to identify a point as a definite projectile armature. A score equal to or higher than 4 is considered sufficient for projectile identification. The higher the score, the more reliable the interpretation is. The scoring criteria presented above are summarized in Table S12.

**Text S4.3 High magnification analysis**

The aim of the analysis was to detect MLITs (microscopic linear impact traces)^7,25,46^ to corroborate projectile identifications and to record hafting-related microwear^47^ to find evidence for or against the hafting configuration proposed for the archaeological points. Of the sample of 16 tanged points and fragments, 15 were analyzed for MLITs and 10 for hafting wear. The microwear on tangs was compared with that documented on 16 experimental points from the samples reported here. These artefacts were hafted with sinew bindings (n=6), glue (n=5), and glue-sinew-glue (n=5). They were all analyzed in detail under low and high magnification before hafting to avoid confusion between production and haft wear, and again after their use.

***MLITs on archaeological points***. Pronounced MLITs were observed on some of the archaeological points with substantial impact damage (Figs. 1b-d and 2c in main text), but they proved to be very infrequent. Obvious examples could be found only on two out of the 15 analyzed projectiles, with a third artefact showing striations that could not be linked to projectile use with full certainty.

***Hafting wear.*** Of the ten archaeological points examined for hafting wear with both low and high magnification, four showed scars on the shoulders or their immediate vicinity, corresponding to damage recorded at haft limit on some experimental points. While these scars might relate to hafting, they did not by themselves allow inferring the hafting configuration. Under high magnification, five projectiles showed occasional light polish on the lateral sides of the tang, but it was not characteristic enough to rule out production and/or taphonomic wear. For four artefacts, the observations were limited due to either taphonomic features or varnish that had been applied on the tangs at some point during the curational history of the artefacts and that could not be fully removed.

The observed microwear did not allow positively confirming the proposed hafting system, but importantly, no contradicting evidence was encountered, either.

The last piece (P359.B2.13) presented a thin, straight tang. Bright spots were recorded dominantly on the dorsal surface and, to a lesser extent, the right lateral edge of the tang around the area where haft limit would have been located on morphological grounds. As the tang is straight in profile, lateral shaft contact would not have been an obligation for hafting this point, and it cannot be ruled out that it represents an alternative hafting configuration. However, bright spots may form also taphonomically^48^. Further, the experimental sample showed that the primary location of shaft contact does not straightforwardly determine the distribution of haft wear.

Due to the inconclusive results obtained on the ten archaeological artefacts, this part of the analysis was not pursued further.

**Text S4.4 RIS analysis**

The fracture grouping employed here builds on the analysis of an extensive experimental dataset (see Text S2) and aimed to find impact fracture categories informative of the RIS characteristic of each weapon system^26^ without compromising sample size. The four prehistoric weapons discussed here are distinct in their angle of incidence^26^, which determines the relative importance of the bending and compressive components of the force that breaks the armature. After exploration of the experimental data^6^, it could be established that the relative frequency of bending-initiated breaks (BB) – regardless of their termination – is a suitable marker for the relative importance of the bending component on impact if all other parameters are held constant. In contrast, the relative proportion of secondary damage (SD) was found to sufficiently reflect the importance of the compressive component. Finally, the relative proportion of LS is characteristic of the length of contact between the lithic point and resistant material (usually bone) in the target, which depends on the mass of the projectile^6^. In grouping different fracture attributes when variation in these attributes is insignificant for the main question (e.g. variation in terminations of breaks initiated by bending) and in focusing on features that occur frequently on experimental projectiles, this classification system maximizes sample sizes while maintaining their information potential, and is therefore suitable for archaeological analysis even when strict projectile identification criteria are applied.

**Text S5 Experimental program**

The ballistic characteristics of prehistoric weapons and their effect on lithic armature breakage on impact can only be studied through replicative experiments. The goal of such experimental programs is to reproduce, under monitored conditions, the fracture patterns observed on the archaeological material, with the aim of identifying the weapon design and delivery system responsible for this fracture pattern. Impact fracture formation is a complex phenomenon driven by the interaction between the stress produced by the weapon delivery method, the reception of this stress by the armature and its hafting arrangement, the morphology of the armature, and the fracture mechanical properties of its raw material. Our experimental program was set up in three main phases. The objective of the first was to see if the hypothesized hafting system worked and if it was able to generate RIS conditions corresponding to the fracture patterns observed in the archaeological material. The second phase was designed to address the question of the weapon system based on the results of the first experiment and to adjust experimental parameters to better match the archaeological setting if necessary. The third phase tested the effect of tang width on the formation of the lateral tang breaks to critically evaluate the earlier results and verify that they were not affected by selection bias. It also served the purpose of increasing the sample size to allow statistical testing.

**Text S5.1 Production of tanged points**

The production of the experimental tanged point replicas necessitated a profound understanding of the shaping techniques and gestures used for their production^6,49^ to copy their formal characteristics as accurately as possible. We followed the *chaîne opératoire* reconstructed for the tanged points by technological analysis^6,50^. We additionally documented the morphometric variability of the archaeological tanged points (n=16) using a set of measurements (Fig. S7).

The replication of the points required significant skill and time investment, with each point taking between 20 and 60 minutes to produce. To achieve the best result, we split the production in two steps and divided the work among four knappers. Manuel Maingeot (with 20 years of knapping experience) and Christian Lepers (23 years of experience) produced most of the blanks. The points were then shaped by Justin Coppe (13 years of experience) and Philippe Pirson (21 years of experience).

The tanged points were produced by successive generations of direct flat retouch. The blank was shaped by retouch according to the need, with some naturally pointed pieces requiring retouch only for the production of the tang. The edges were prepared by abrading with a sandstone hammer and the flat retouch applied by marginal percussion with an antler hammer. Two varieties of flint were used for replication: Harmignies flint from the Spiennes quarry in Belgium and flint from the Haubourdin quarry in France.

**Text S5.2 First projectile experiment**

***Goals and setup.*** The morphology of the tanged points from Maisières-Canal and the RIS analysis results suggested a hafting arrangement where shaft contact was with the lateral sides of the tang (Figs. S2, S8). The first experiment was designed to test whether this solution could produce the archaeological fracture patterns, including the laterally initiated tang breaks (Fig. 1). As a general rule, the stronger the hafting system is, the more probable is the breakage of the lithic implement on impact. We tested three binding systems of varying strengths to determine which level of resistance was needed to create the BB on the tang. The 30 points produced for the first experiment by respecting the morphometrics and shaping sequence of their archaeological counterparts (Text S4.1) were fitted with an axial orientation in split wooden shafts with the sides of the tang in contact with the wood. Ten points were lashed on their shafts with horse sinew bindings. Another ten were secured with a mix of beeswax and spruce resin (30% and 70% of mass, respectively). The remaining ten points were fixed with the same adhesive mix, bound with horse sinew after that, and the bindings finally covered with the adhesive to protect and reinforce the bond (Fig. S3).

An artificial target was constructed for the experiment, composed of 250 kg of 10% ballistic gel in which a pony skeleton was set in anatomical position. The gel was shaped to mimic animal anatomy and covered with a stretched, rehydrated, 1–1.5 mm thick deer skin following a protocol described previously^32,51^. The target was originally designed to model a horse to match the results of the first faunal study on Maisières-Canal^52^, but in view of recent data that recognized reindeer as the best-represented large game at the site^53^, a deer skin was finally opted for. As the new faunal data came in when the preparations for our experiment had already begun, we were unable to obtain a complete deer skeleton in the set time frame, and the target therefore represents a compromise.

Of the 30 points, three were shot with a bow and nine points were launched with each of the other classic propulsion modes (spearthrower, throwing and thrusting spear). The projectiles were shot until visible damage occurred. In case of none, the experiment was stopped after ten shots. After each shot, the target was carefully examined before the weapon was retrieved to record which bones (if any) the flint point had impacted. Bow and spearthrower shots were fired from a 10 m distance, hand cast spears from a 5 m distance, and thrusting spears were launched from the immediate vicinity of the target. The bow and spearthrower shots were taken by C. Lepers. The bow used was a replica of a Neolithic longbow, made from yew and with a draw weight of 48 pounds at 29-inch draw length. Throwing and thrusting spear shots were taken by J. Coppe with replicas of the wooden spears from Schöningen^54,55^, made from spruce. The gestures used correspond to those reported in previous works^26,27^. The physical characteristic of the shaft used during this experiment is described in Table S8.

***Results***. The hafting design (Fig. S2b, S8) tested in this experiment proved perfectly efficient. All the weapon delivery methods achieved penetration depths sufficient to inflict a mortal wound by bleeding (Fig. S4). Only two glue-hafted points, one secured on an arrow and one on a throwing spear, failed to penetrate the deer skin and de-hafted upon impact. Out of the 30 experimental points, 19 points fractured in use, resulting in a total of 60 fractures. Of the remaining 11 points, four missed the target and hit the wall (these points were lost), while seven others remained undamaged.

We succeeded in producing a BB on a tang, initiated on its right lateral edge (Fig. S5). This break is associated with LS located on the right lateral side of the limb. This fracture pattern was observed on a dart point that was secured with a glue-sinew-glue (GSG) bond and hit the scapula. The fracture is identical to what was observed archaeologically, and its presence demonstrates that the experimental parameters we chose approached the archaeological conditions. We did not, however, succeed in reproducing the exact fracture signature of the archaeological sample (Fig. S9, Table S13), as SD are absent in the experimental set.

***Evaluation***. While we cannot guarantee that the hafting system we used is an exact match to the archaeological points, the experiment confirmed that the fracture pattern observed archaeologically minimally requires the same contact surface between the edges of the tangs and the shaft (i.e., lateral contact) and a similar strength to the GSG solution we used in the experiment. We could therefore consider the hafting system to be sufficiently replicated and proceed to attempting to identify the weapon delivery system.

Several factors could explain the absence of SD in the first experimental set. The experimental points were slightly thicker and wider than the archaeological points, and their edge angles somewhat less acute (Fig. S10, Table S14). This could make the experimental points more resistant to fracturing than the archaeological ones. Also point raw materials differed to an extent: the archaeological points were made from Obourg flint which is generally more fine-grained than the Harmignies flint that was available to us for experimental replication (the natural outcrops of Obourg flint being nearly exhausted). The absence of SD on the experimental points could be linked to this difference in raw material quality and to the overall robustness of the experimental points compared to the archaeological ones. These discrepancies were addressed in the second experiment.

**Text S5.3 Second projectile experiment**

***Goals and setup***. The morphology of the experimental points was now more closely monitored to improve the morphometric similarity between the experimental and archaeological points (Fig. S11, Table S15). A finer grained flint closer in its qualities to the Obourg flint was obtained from the Haubourdin quarry (France) on the advice of Jean-Phillipe Collin. The target was also adapted by replacing the pony skeleton with a deer skeleton. As the strongest binding system (GSG) used in the first experiment appeared to produce the closest match with archaeological fracture patterns, all the points in the second experiment were hafted with this system. Nine points were shot per weapon system, producing a subsample of 36 points.

***Results***. Out of 36 points shot at the target, 28 points presented impact damage, amounting to a total of 75 fractures. SD are now present in the experimental sample, and the specific fracture pattern with a BB initiated on a lateral side of a tang and associated with LS on the same side of the limb could again be produced on two pieces. Once more, this fracture pattern only occurred when the spearthrower was used and the point hit either the scapula or the spine. This reinforces the link between the spearthrower and the Maisières-Canal fracture signal. The second closest match is with the throwing spear, whereas the fracture signals of the bow and thrusting spear are notably different (Fig. S12, Table S16). However, while highly similar, the fracture proportions achieved with the spearthrower are still not a perfect match with the archaeological sample. The relative frequency of SD is still a bit too low, and the LS are slightly overrepresented (Fig. S12, Table S16).

***Evaluation.*** The remaining difference between the experimental spearthrower sample and the archaeological sample could be affected by sample size. The experimental sample contains 16–21 fractures for each mode of propulsion, whereas the archaeological count is 62. In addition, a possible source of bias was detected during the analysis of the experimental material. Archaeologically, the BB initiated on the lateral side of a tang were always observed on tangs with a maximum width of 12 mm. In our experimental sample, no attention was paid to distributing different tang widths across the subsamples (thrusting spear, throwing spear, spearthrower, bow) equally. As arrows and darts generally require rather narrow tangs to be hafted efficiently, this led to an overrepresentation of tangs with a maximum width of 12 mm within the arrow and dart samples and their underrepresentation among the thrusting and throwing spears. We decided to carry out a supplementary experiment to test whether the absence of the characteristic tang breaks in the spear sample might be due to this bias and to increase the size of the experimental sample.

**Text S5.4 Third projectile experiment**

***Goals and setup.*** The third experiment involved only three weapon delivery methods (spearthrower, throwing and thrusting spear), as the suspected bias did not concern arrowheads. We produced tanged pieces that matched the morphometrics of the archaeological sample (Fig. S13, Table S17) with a maximum tang width of 12 mm and mounted them using the glue-sinew-glue solution. During this experiment we specifically aimed for the toughest part of the skeleton (the scapula and the spine) to maximize the chances of tang breakage with all modes of propulsion.

***Results.*** Out of the 33 pieces shot during this experiment, one was lost and three missed the target and impacted its wooden base. In total, 29 pieces successfully hit the target and displayed 101 fractures. Once again, we succeeded in reproducing the specific fracture pattern with a BB initiated on a lateral side of a tang and associated with LS on the same edge of the limb in the distal part on two points, again shot with the spearthrower. Two thrusting spear points also broke at the tang, but the breaks were dorsally or ventrally initiated and no LS were present on the limb.

***Evaluation***. This experiment permitted eliminating the bias present in the earlier experimental sample. We could conclude that the characteristic fracture pattern observed on the archaeological points (laterally initiated tang break associated with lateral scarring on the limb) can only be reproduced with the spearthrower. The experimental spearthrower fracture signal is the closest match for the archaeological signal (Fig. S14, Table S18).

Supporting Information Figures S1-S14


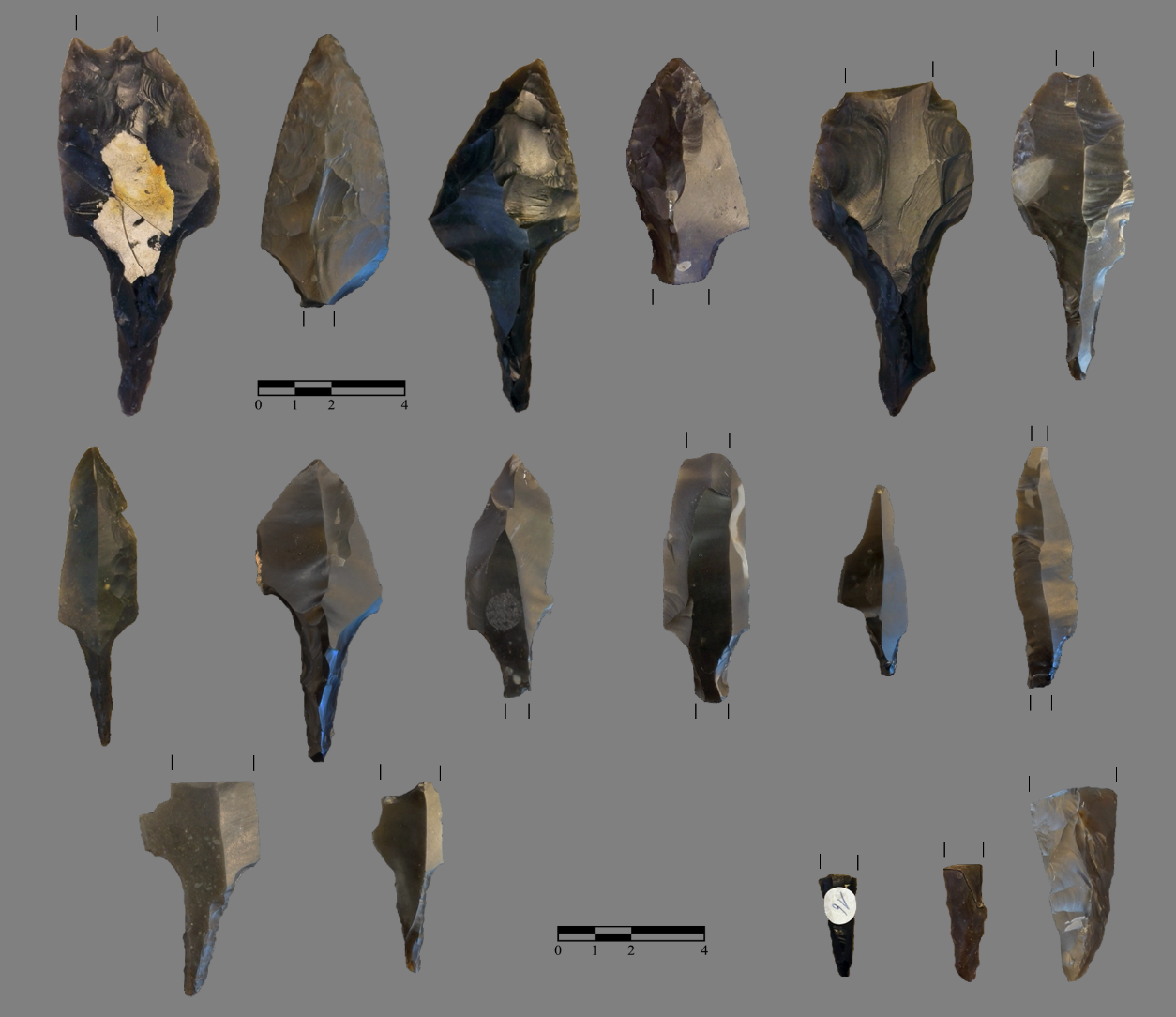


**Figure S1.** Dorsal views of artefacts identified reliably as projectiles.


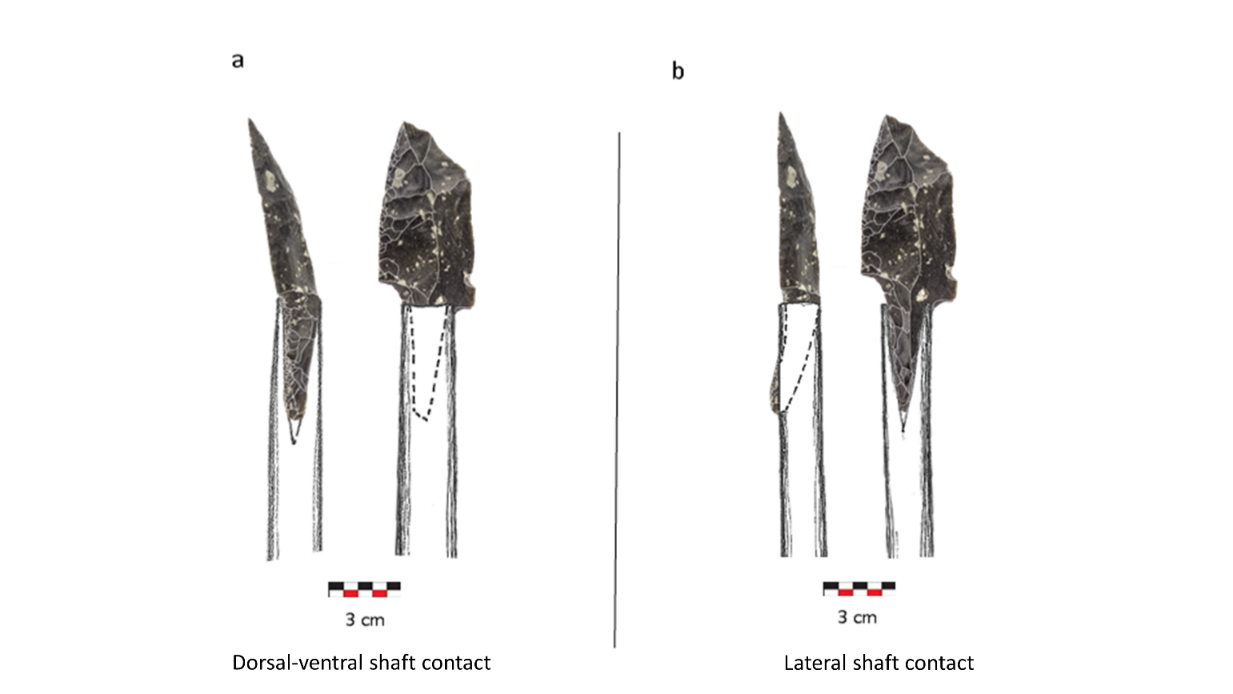


**Figure S2**. Two alternative hafting configurations, illustrating the misalignment between the point and the shaft if contact was on the dorsal and ventral surfaces of the tang (a) and the lateral contact configuration that mends the problem (b). Artefact photo by O. Touzé^56^.


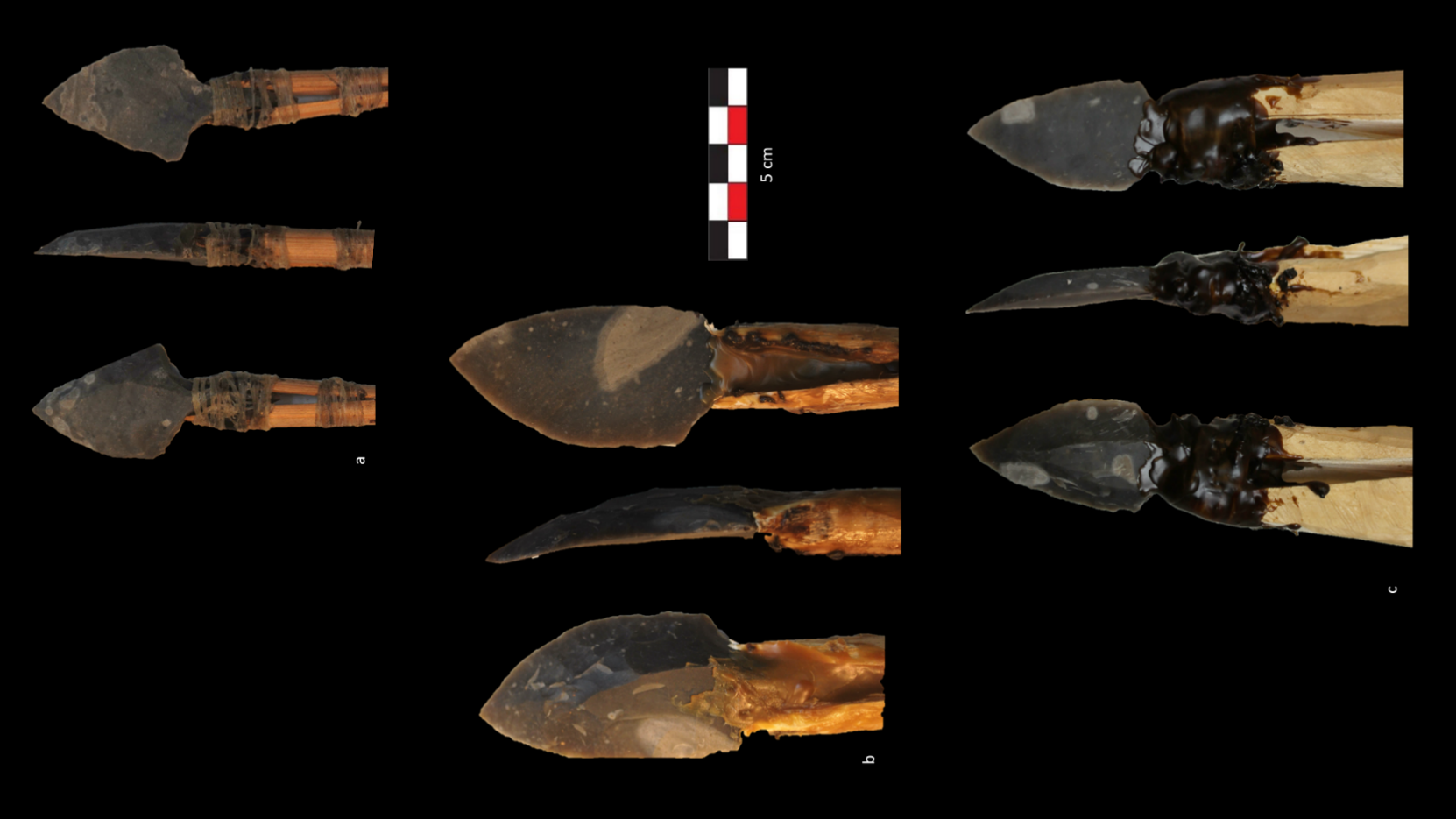


**Figure S3.** Different bonding techniques used in the first projectile experimental. a: Sinew bindings on an arrow; b: Spruce resin-beeswax glue on a dart; c: Glue-sinew-glue (GSG) on a spear.

**Figure S4**. Boxplot showing the depth of penetration (in cm) for different hafting systems and weapon delivery systems. Data in Table S19.


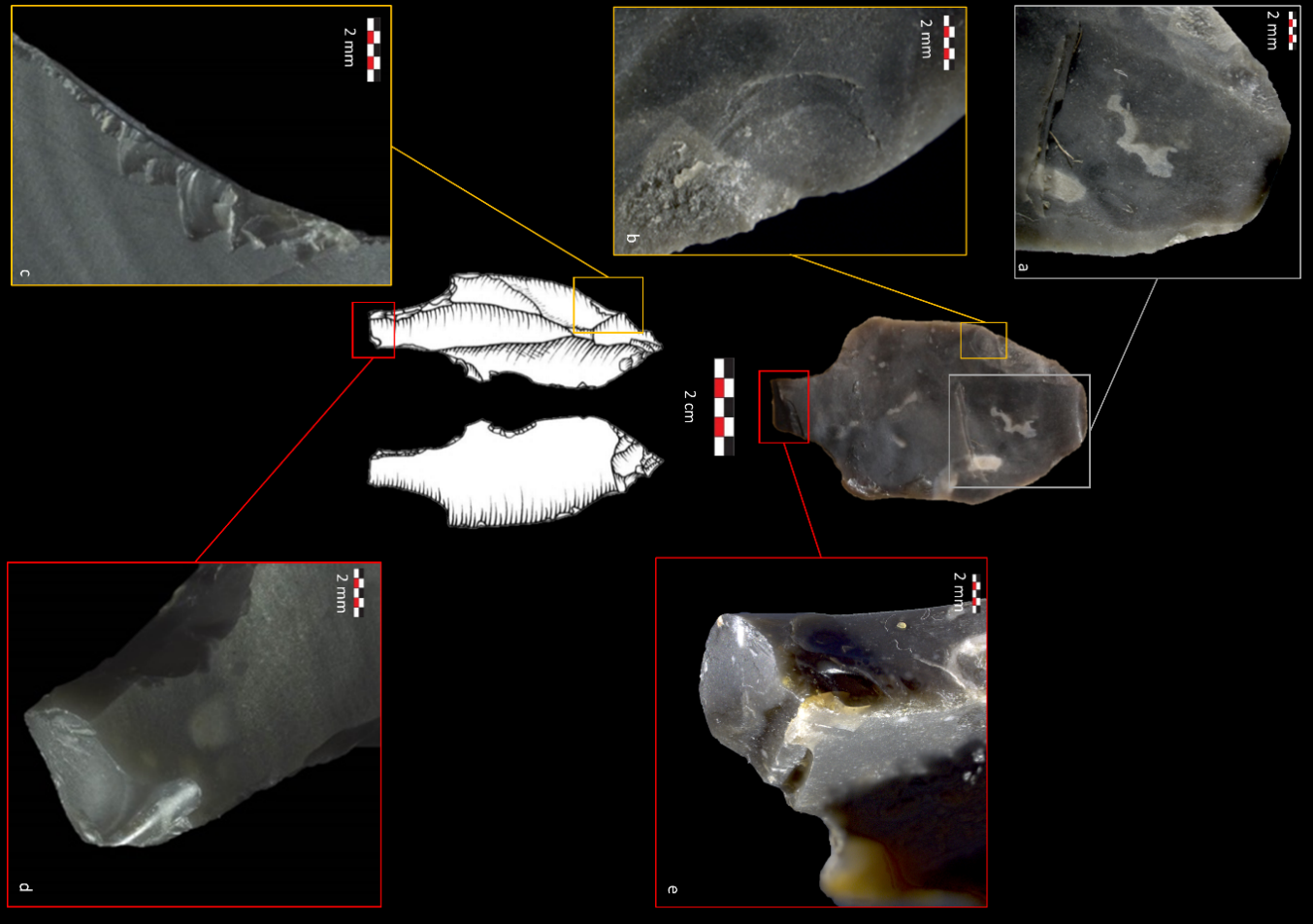


**Figure S5.** Comparison between fracture pattern between an experimental tanged point (top) and the archaeological point P356.B6.1 (bottom). Both pieces present a similar pattern with LS (single in b and multiple in c) located on the edge where the BB on the tang was initiated


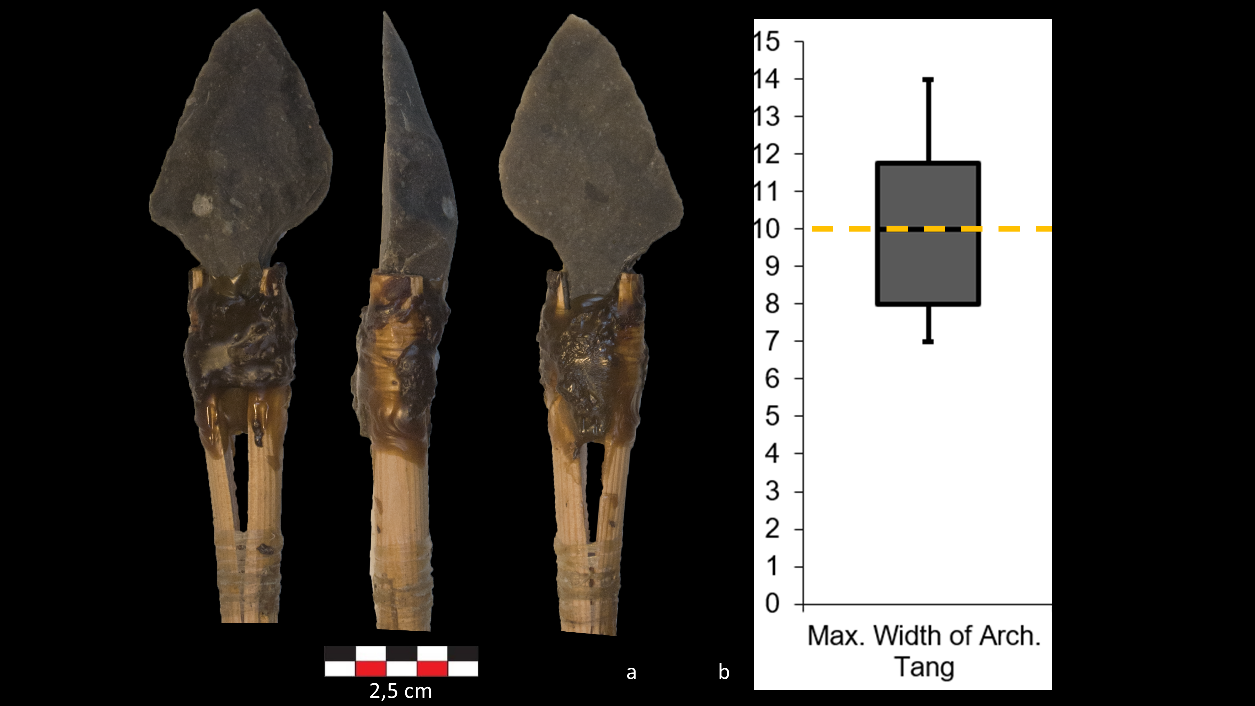


**Figure S6.** a. Experimental point with a 10 mm wide tang hafted in an 8 mm shaft, representing maximum tang width for arrows. b. Boxplot showing tang width for the archaeological tanged points identified as projectiles.


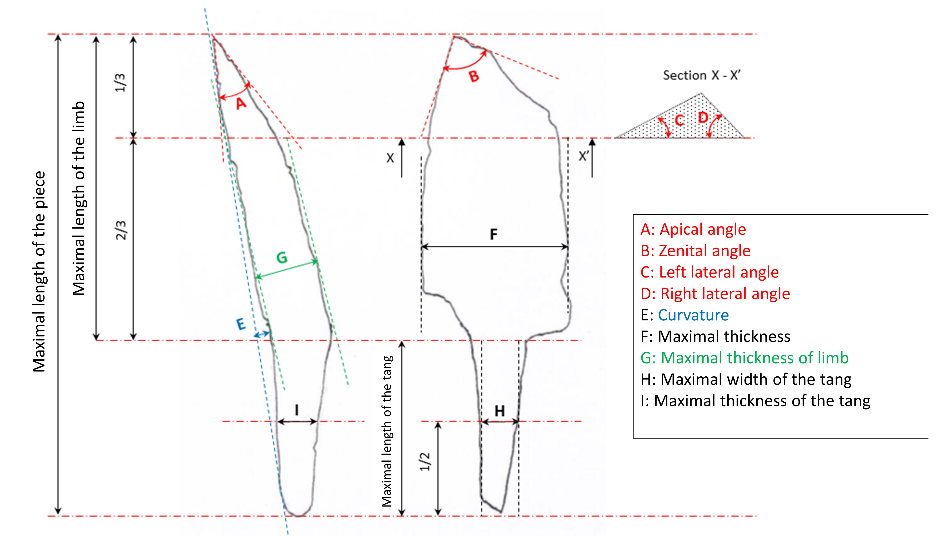


**Figure S7.** Measurements taken on the archaeological points to aid experimental replication.


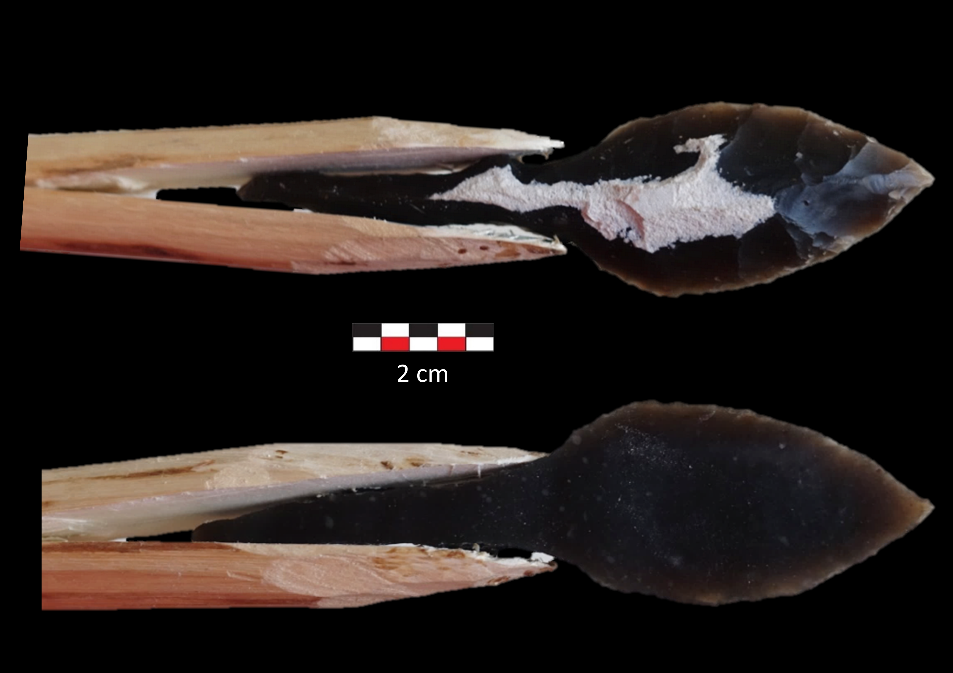


**Figure S8**. Experimental tanged point in a split wooden shaft with contact on the lateral sides of the tang.


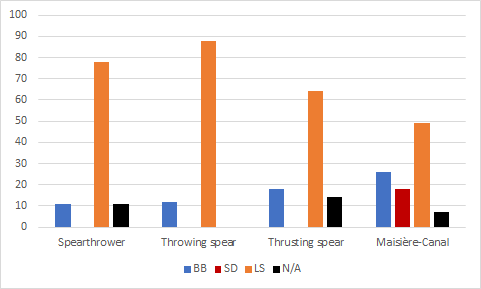


**Figure S9.** Fracture signals (in %) obtained for each weapon delivery system in the first experiment, compared with the Maisières-Canal sample (data in Table S13).

**Figure S10.** Morphometric comparison of the replicas used in the first experiment and the archaeological tanged point sample (data in Table S14).

**Figure S11.** Morphometric comparison of the replicas used in the second experiment and the archaeological tanged point sample (data in Table S15).


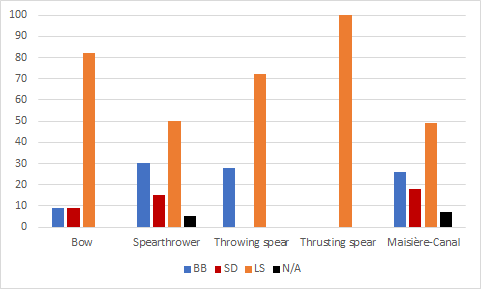


**Figure S12**. Fracture signals (in %) obtained for each weapon delivery system in the second experiment, compared with the Maisières-Canal sample (data in Table S16).

**Figure S13.** Morphometric comparison of the replicas used in the third experiment and the archaeological tanged point sample (data in Table S17).


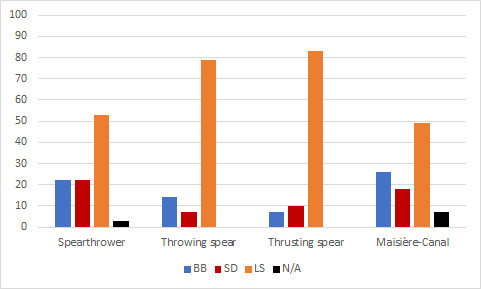


**Figure S14.** Fracture signals (in %) obtained for each weapon delivery system in the third experiment, compared with the Maisières-Canal sample (data in table S18).

Supporting Information Tables S1-S19

**Table S1**. Typological breakdown of the retouched tool assemblage from Maisières-Canal.

| Tool type | n | % |
| --- | --- | --- |
| Maisières points | 121 | 12.8 |
| Tanged points | 56 | 6 |
| Distal point fragments | 20 | 2.1 |
| Pointed retouched blades | 9 | 1 |
| Shouldered points | 8 | 0.8 |
| *Total points* | *214* | *22.6* |
| Burins | 382 | 40.4 |
| Retouched blades | 123 | 13 |
| Other tanged pieces | 83 | 8.8 |
| Scrapers | 49 | 5.2 |
| Retouched flakes | 42 | 4.4 |
| Perforators | 15 | 1.6 |
| Combination tools | 11 | 1.2 |
| Other shouldered pieces | 9 | 1 |
| Indeterminate fragments | 8 | 0.8 |
| Notched pieces | 4 | 0.4 |
| Backed pieces | 3 | 0.3 |
| Denticulates | 2 | 0.2 |
| Total | 945 | 100 |

Data from^50^.

**Table S2.** Count and relative proportion of each fracture category in the sample of archaeological points identified as projectile armatures.

|  | Tanged point (n=17) | | Maisières points (n=11) | |
| --- | --- | --- | --- | --- |
|  | n fract. | % | n fract. | % |
| *BB* | 17 | 26 | 11 | 26 |
| *SD* | 12 | 18 | 9 | 22 |
| *LS* | 32 | 49 | 21 | 50 |
| N/A | 5 | 8 | 1 | 2 |
| Total fractures | 66 | 100 | 42 | 100 |

The four distal fragments are not listed because they could not be assigned to a typological category.

**Table S3**. Results of the χ2 test between the experimental sample of point used with spear-thrower from the second and third projectile experiment and the archaeological sample.

| Tests χ² | | | |
| --- | --- | --- | --- |
|  | Value | df | p value |
| χ² | 0,100 | 2 | 0,951 |
| n | 107 |  |  |

Sample data in Table S6.

**Table S4.** Results of the χ2 test between the experimental sample of point used with thrusting spear from the second and third projectile experiment and the archaeological sample.

| Tests χ² | | | |
| --- | --- | --- | --- |
|  | Value | df | p value |
| χ² | 17,6 | 2 | < 0,001 |
| n | 114 |  |  |

Sample data in Table S6.

**Table S5.** Results of the χ2 test between the experimental sample of point shot with throwing spear from the second and third projectile experiment and the archaeological sample.

| Tests χ² | | | |
| --- | --- | --- | --- |
|  | Value | df | p value |
| χ² | 7.05 | 2 | 0,029 |
| n | 101 |  |  |

Sample data in Table S6.

**Table S6.** Sample for the χ2 test comparing experimental and archaeological fracture signals.

|  | Experimental tanged points | | | | | | Archaeological tanged points | |
| --- | --- | --- | --- | --- | --- | --- | --- | --- |
|  | Spearthrower (n=16) | | Throwing spear (n=17) | | Thrusting spear (n=16) | | n=17 | |
|  | n fract. | % | n fract. | % | n fract. | % | n fract. | % |
| *BB* | 13 | 26 | 9 | 20 | 3 | 5 | 17 | 28 |
| *SD* | 10 | 20 | 2 | 4 | 4 | 7 | 12 | 20 |
| *LS* | 27 | 54 | 35 | 76 | 50 | 88 | 32 | 53 |
| Total fract. | 50 | 100 | 46 | 100 | 57 | 100 | 61 | 100 |

Result from the second and the third experiment without the bow sample and the N/A fracture categories that does not meet the minimum sample size for a χ2 test.

**Table S7.** Result of fracture proportion from the second and third experiment divided by mode of propulsion (data for Fig. 3).

|  | Experimental tanged points | | | | | | | | Archaeological tanged points | |
| --- | --- | --- | --- | --- | --- | --- | --- | --- | --- | --- |
|  | Bow (n=8) | | Spearthrower (n=16) | | Throwing  spear  (n=17) | | Thrusting spear  (n=16) | | n=17 | |
|  | n fract. | % | n fract. | % | n fract. | % | n fract. | % | n fract. | % |
| *BB* | 2 | 9 | 13 | 25 | 9 | 20 | 3 | 5 | 17 | 26 |
| *SD* | 2 | 9 | 10 | 19 | 2 | 4 | 4 | 7 | 12 | 18 |
| *LS* | 17 | 82 | 27 | 52 | 35 | 76 | 50 | 88 | 32 | 49 |
| N/A | 0 | 0 | 2 | 4 | 0 | 0 | 0 | 0 | 5 | 8 |
| Total fract. | 21 | 100 | 52 | 100 | 46 | 100 | 57 | 100 | 66 | 100 |

**Table S8.** Technical details for the experimental projectile shafts.

|  | Min | Median | Max |
| --- | --- | --- | --- |
| Arrow spine (cm) | 1,1 | 1,2 | 1,5 |
| Dart spine (cm) | 3 | 4,6 | 7,2 |
| Arrow weight (g) | 45 | 48 | 59 |
| Dart weight (g) | 160 | 208 | 311 |
| Spear weight (g) | 497 | 600 | 750 |
| Arrow length (cm) | 80 | | |
| Dart length (cm) | 210 | | |
| Spear length (cm) | 210 | | |

Weights include the lithic point. Spine measured as in^32^. The arrow shafts were made from pine, the dart shafts from hazelnut, and the spear shafts from spruce.

**Table S9**. TCSA metrics for the archaeological tanged points identified as spearthrower tips (data for Fig. 4).

| n | 13 |
| --- | --- |
| Mean | 164 |
| Median | 145 |
| Standard deviation | 90,2 |
| Minimum | 45 |
| Maximum | 323 |

**Table S10.** Typological breakdown of the archaeological samples.

| Typological category | Screened material | Sample for detailed analysis | Identified as projectile armatures |
| --- | --- | --- | --- |
| Maisières points | 121 | 32 | 11 |
| Tanged points | 56 | 31 | 14 |
| Point fragments | 20 | 10 | 4 |
| Tang fragments | 21 | 16 | 3 |
| Backed pieces | 3 | 0 | 0 |
| Shouldered points | 8 | 2 | 0 |
| Unretouched blades | 100 | 5 | 0 |
| Total | 329 | 97 | 31 |

**Table S11**. Features recorded on the pieces identified as projectile armatures, compared with the rest of the sample.

|  | | | **Pieces identified as projectile armatures (n=32)** | | **Remaining sample (n=66)** | |
| --- | --- | --- | --- | --- | --- | --- |
|  |  |  | n fractures | n pieces | n fractures | n pieces |
| BB | Termination morphology | Snap | 12 | 10 | 16 | 14 |
|  |  | Abrupt | 12 | 12 | 15 | 15 |
|  |  | Feather | 9 | 9 | 6 | 6 |
|  |  | Indeterminate | 0 | 0 | 2 | 1 |
|  | Fissured termination | Yes | 1 | 1 | 2 | 2 |
|  |  | No | 33 | 25 | 37 | 31 |
|  | MLITs | | 0 | 0 | NA | NA |
|  | Possible MLITs | | 4 | 4 | NA | NA |
| Total BB | | | 33 | / | 39 | / |
| SD | Fracture composition | Single | 7 | 4 | 5 | 5 |
|  |  | Multiple | 20 | 14 | 1 | 1 |
|  | Initiation morphology | Absent | 16 | 10 | 3 | 2 |
|  |  | Present (cone/ bending/ intermediate) | 14 | 11 | 3 | 3 |
|  | Fissured  termination | Yes | 8 | 6 | 0 | 0 |
|  |  | No | 19 | 12 | 6 | 5 |
|  | Location of termination | Same than their BB | 10 | 8 | 4 | 4 |
|  |  | Opposite | 17 | 8 | 2 | 1 |
|  | MLITs | | 1 | 1 | NA | NA |
|  | Possible MLITs | | 0 | 0 | NA | NA |
| Total SE | | | 29 | / | 4 | / |
| LS | Fracture composition | Single | 31 | 16 | 18 | 14 |
|  |  | Multiple | 33 | 22 | 11 | 8 |
|  | Initiation morphology | Absent | 25 | 15 | 12 | 9 |
|  |  | Present (cone/ bending/ intermediate) | 43 | 24 | 21 | 17 |
|  | Fissured termination | Yes | 21 | 11 | 0 | 0 |
|  |  | No | 43 | 18 | 29 | 18 |
|  | General direction | Diagonal | 45 | 24 | 0 | 0 |
|  |  | Mixed | 0 | 0 | 16 | 12 |
|  |  | Perpendicular | 19 | 10 | 13 | 10 |
|  | MLITs | | 3 | 1 | NA | NA |
|  | Possible MLITs | | 3 | 3 | NA | NA |
| Total LS | | | 64 | / | 29 | / |
| Total Indeterminate | | | 7 | / | 3 | / |
| Total fractures | | | 133 |  | 77 |  |

**Table S12.** Synthesis of the points given to each potential projectile depending on the morphological attributes of fractures. *BB with snap termination are not counted.

|  | Present* | Fissured termina-tion | Length respect-tive to the cross-section of the fracture | Multiple | Re-moved initiation | Located on the surface of BB initiation | Initiated on the abrupt termina-tion of the primary break | Maxi-mum value |
| --- | --- | --- | --- | --- | --- | --- | --- | --- |
| *BB* | +1 | +1 | +1 | / | / | / | / | 3 |
| *SE* | +1 | +1 | +1 | +1 | +1 | +1 | +1 | 7 |
| *LS* | +1 | +1 | +1 | +1 | +1 | / | / | 5 |

**Table S13.** Fracture proportions in the first experimental set (all binding systems included) for each weapon delivery system, compared with the archaeological data (data for Fig. S9).

|  | Experimental tanged points | | | | | | | | Archaeological tanged points | |
| --- | --- | --- | --- | --- | --- | --- | --- | --- | --- | --- |
|  | Bow (n=2) | | Spearthrower (n=4) | | Throwing spear  (n=5) | | Thrusting spear  (n=8) | | n=17 | |
|  | n fract. | % | n fract. | % | n fract. | % | n fract. | % | n fract. | % |
| *BB* | 2 | / | 2 | 11 | 2 | 12 | 4 | 18 | 17 | 26 |
| *SD* | 0 | / | 0 | 0 | 0 | 0 | 0 | 0 | 12 | 18 |
| *LS* | 1 | / | 14 | 78 | 15 | 88 | 15 | 68 | 32 | 49 |
| N/A | 0 | 0 | 2 | 11 | 0 | 0 | 3 | 14 | 5 | 8 |
| Total fract. | 3 | / | 18 | 100 | 17 | 100 | 22 | 100 | 66 | 100 |

**Table S14.** Comparison of morphometrics of the replicas used in the first experiment and the archaeological tanged point sample (data for Fig. S10).

|  | Max. Exp Width | Max. Arch. Width | Max. Exp. Thick | Max. Arch. Thick | Exp Ap. Angle | Arch. Ap. Angle | Exp. Lat. Angle | Arch. Lat. Angle | Exp. Zen. Angle | Arch. Zen. Angle |
| --- | --- | --- | --- | --- | --- | --- | --- | --- | --- | --- |
| Min | 25,3 | 18,0 | 7,4 | 5,0 | 21,0 | 19,0 | 20,0 | 22,0 | 44,0 | 24,0 |
| Q_1_ | 31,0 | 23,8 | 10,6 | 6,8 | 25,0 | 23,0 | 36,0 | 27,0 | 49,3 | 54,3 |
| Median | 34,7 | 31,5 | 11,4 | 10,5 | 31,5 | 26,5 | 44,0 | 33,0 | 56,5 | 69,0 |
| Q_3_ | 39,4 | 35,8 | 12,8 | 13,3 | 36,0 | 29,3 | 46,0 | 35,3 | 65,8 | 86,0 |
| Max | 45,3 | 45,0 | 16,0 | 15,0 | 48,0 | 32,0 | 65,0 | 48,0 | 80,0 | 94,0 |
| IQR | 8,4 | 12,0 | 2,2 | 6,5 | 11,0 | 6,3 | 10,0 | 8,3 | 16,5 | 31,8 |
| Upper Outliers | 0 | 0 | 0 | 0 | 0 | 0 | 1 | 1 | 0 | 0 |
| Lower Outliers | 0 | 0 | 0 | 0 | 0 | 0 | 1 | 0 | 0 | 0 |

Max.=maximum; Exp.=experimental point; Arch.=archaeological point; Thick.=thickness; Ap.=apical; Lat.=lateral; Zen.=zenital; see Fig. S6.

**Table S15.** Comparison between the morphometrics measurement between the experimental reproductions for the second experiment and the archaeological tanged point sample (data for Fig. S11).

|  | Max. Exp. Width | Max. Arch. Width | Max. Exp. Thick. | Max. Arch. Thick. | Exp. Ap. Angle | Arch. Ap. Angle | Exp. Lat. Angle | Arch. Lat. Angle | Exp. Zen. Angle | Arch. Zen. Angle |
| --- | --- | --- | --- | --- | --- | --- | --- | --- | --- | --- |
| Min | 27,2 | 18,0 | 7,1 | 5,0 | 26,0 | 19,0 | 30,0 | 22,0 | 47,0 | 24,0 |
| Q_1_ | 30,9 | 23,8 | 9,3 | 6,8 | 31,5 | 23,0 | 35,8 | 27,0 | 64,3 | 54,3 |
| Median | 33,1 | 31,5 | 10,0 | 10,5 | 34,0 | 26,5 | 39,0 | 33,0 | 74,5 | 69,0 |
| Q_3_ | 38,6 | 35,8 | 11,9 | 13,3 | 39,0 | 29,3 | 42,0 | 35,3 | 79,0 | 86,0 |
| Max | 43,6 | 45,0 | 14,6 | 15,0 | 65,0 | 32,0 | 50,0 | 48,0 | 83,0 | 94,0 |
| IQR | 7,7 | 12,0 | 2,5 | 6,5 | 7,5 | 6,3 | 6,3 | 8,3 | 14,8 | 31,8 |
| Upper Outliers | 0 | 0 | 0 | 0 | 1 | 0 | 0 | 1 | 0 | 0 |
| Lower Outliers | 0 | 0 | 0 | 0 | 0 | 0 | 0 | 0 | 0 | 0 |

Max=maximum; Exp.=experimental point; Arch.=archaeological point; Thick.=thickness; Ap.=apical; Lat.=lateral; Zen.=zenital; see Fig. S6.

**Table S16.** Fracture proportions in the second experimental set for each weapon delivery system, compared with the archaeological data (data for Fig. S12).

|  | Experimental tanged points | | | | | | | | Archaeological tanged points | |
| --- | --- | --- | --- | --- | --- | --- | --- | --- | --- | --- |
|  | Bow (n=8) | | Spearthrower (n=7) | | Throwing spear  (n=7) | | Thrusting spear  (n=6) | | n=17 | |
|  | n fract. | % | n fract. | % | n fract. | % | n fract. | % | n fract. | % |
| *BB* | 2 | 9 | 6 | 30 | 5 | 28 | 0 | 0 | 17 | 26 |
| *SD* | 2 | 9 | 3 | 15 | 0 | 0 | 0 | 0 | 12 | 18 |
| *LS* | 17 | 82 | 10 | 50 | 13 | 72 | 16 | 100 | 32 | 49 |
| N/A | 0 | 0 | 1 | 5 | 0 | 0 | 0 | 0 | 5 | 8 |
| Total fract. | 21 | 100 | 20 | 100 | 18 | 100 | 16 | 100 | 66 | 100 |

**Table S17.** Comparison between the morphometrics measurement between the experimental reproductions for the third experiment and the archaeological tanged point sample (data for Fig. S13).

|  | Max. Exp. Width | Max. Arch. Width | Max. Exp. Thick. | Max. Arch.Thick. | Exp. Ap. Angle | Arch. Ap. Angle | Exp. Lat. Angle | Arch. Lat. Angle | Exp. Zen. Angle | Arch. Zen. Angle |
| --- | --- | --- | --- | --- | --- | --- | --- | --- | --- | --- |
| Min | 23,1 | 18,0 | 5,6 | 5,0 | 22,0 | 19,0 | 24,0 | 22,0 | 60,0 | 24,0 |
| Q_1_ | 28,7 | 23,8 | 8,3 | 6,8 | 24,0 | 23,0 | 30,0 | 27,0 | 67,5 | 54,3 |
| Median | 31,5 | 31,5 | 9,5 | 10,5 | 27,5 | 26,5 | 33,0 | 33,0 | 75,0 | 69,0 |
| Q_3_ | 35,4 | 35,8 | 10,6 | 13,3 | 31,3 | 29,3 | 36,0 | 35,3 | 81,5 | 86,0 |
| Max | 39,5 | 45,0 | 14,7 | 15,0 | 37,0 | 32,0 | 42,0 | 48,0 | 95,0 | 94,0 |
| IQR | 6,7 | 12,0 | 2,3 | 6,5 | 7,3 | 6,3 | 6,0 | 8,3 | 14,0 | 31,8 |
| Upper outliers | 0 | 0 | 2 | 0 | 0 | 0 | 0 | 1 | 0 | 0 |
| Lower outliers | 0 | 0 | 0 | 0 | 0 | 0 | 0 | 0 | 0 | 0 |

Max=maximum; Exp.=experimental point; Arch.=archaeological point; Thick.=thickness; Ap.=apical; Lat.=lateral; Zen.=zenital; see Fig. S6.

**Table S18.** Fracture proportions in the third experimental set for each weapon delivery system, compared with the archaeological data (data for Fig. S14).

|  | Experimental tanged points | | | | | | Archaeological tanged points | |
| --- | --- | --- | --- | --- | --- | --- | --- | --- |
|  | Spearthrower (n=9) | | Throwing spear  (n=10) | | Thrusting spear  (n=10) | | n=17 | |
|  | n fract. | % | n fract. | % | n fract. | % | n fract. | % |
| *BB* | 7 | 22 | 4 | 14 | 3 | 7 | 17 | 26 |
| *SD* | 7 | 22 | 2 | 7 | 4 | 10 | 12 | 18 |
| *LS* | 17 | 53 | 22 | 79 | 34 | 83 | 32 | 49 |
| N/A | 1 | 3 | 0 | 0 | 0 | 0 | 5 | 8 |
| Total fract. | 32 | 100 | 28 | 100 | 41 | 100 | 66 | 100 |

**Table S19.** Penetration depth (cm) measured for experimental projectiles hafted with different binding systems and launched with different delivery methods.

|  | Glue-sinew-glue | Glue | Sinew | Bow | Spear-thrower | Throwing spear | Thrust-ing spear |
| --- | --- | --- | --- | --- | --- | --- | --- |
| Min | 9,3 | 0,0 | 10,7 | 0,0 | 13,8 | 0,0 | 15,5 |
| Q_1_ | 15,8 | 8,8 | 14,0 | 8,9 | 16,3 | 9,5 | 24,2 |
| Median | 17,5 | 16,1 | 15,8 | 12,2 | 17,0 | 10,9 | 35,5 |
| Q_3_ | 35,1 | 18,9 | 17,1 | 13,5 | 18,5 | 14,3 | 57,5 |
| Max | 63,0 | 44,5 | 65,0 | 16,5 | 20,5 | 17,1 | 65,0 |
| IQR | 19,4 | 10,2 | 3,1 | 4,7 | 2,2 | 4,9 | 33,3 |
| Upper outliers | 0 | 1 | 2 | 0 | 0 | 0 | 0 |
| Lower outliers | 0 | 0 | 0 | 1 | 0 | 1 | 0 |

**SI References**

1. Shea, J. J. The origins of lithic projectile point technology: Evidence from Africa, the Levant, and Europe. *J. Archaeol. Sci.* **33**, 823–846 (2006).

2. Clarkson, C. Testing Archaeological Approaches to Determining Past Projectile Delivery Systems Using Ethnographic and Experimental Data. in *Multidisciplinary Approaches to the Study of Stone Age Weaponry* (eds. Iovita, R. & Sano, K.) 189–201 (Springer, 2016).

3. Newman, K. & Moore, M. W. Ballistically anomalous stone projectile points in Australia. *J. Archaeol. Sci.* **40**, 2614–2620 (2013).

4. Hutchings, W. K. When is a point a projectile? Morphology, impact fractures, scientific rigor, and the limits of inference. in *Multidisciplinary Approaches to the Study of Stone Age Weaponry* (eds. Iovita, R. & Sano, K.) 3–12 (Springer Science+Business Media, 2016). doi:10.1007/978-94-017-7602-8_1.

5. Pétillon, J.-M. *et al.* Hard core and cutting edge: Experimental manufacture and use of Magdalenian composite projectile tips. *J. Archaeol. Sci.* **38**, 1266–1283 (2011).

6. Coppe, J. Sur les traces de l’armement préhistorique : mise au point d’une méthode pour reconstruire les modes d’emmanchement et de propulsion des armatures lithiques par une approche expérimentale, mécanique et balistique. (Université de Liège, 2020).

7. Fischer, A., Vemming Hansen, P. & Rasmussen, P. Macro and micro wear traces on lithic projectile points: experimental results and prehistoric examples. *J. Danish Archaeol.* **3**, 19–46 (1984).

8. Moss, E. H. & Newcomer, M. H. Reconstruction of Tool Use at Pincevent: Microwear and Experiments. in *Tailler! Pourquoi faire: Préhistoire et technologie lithique II, Recent Progress in Microwear Studies. Studia Praehistorica Belgica Leuven 2* 289–312 (1982).

9. Barton, R. N. E. & Bergman, C. A. Hunters at Hengistbury: Some evidence from experimental archaeology. *World Archaeol.* **14**, 237–248 (1982).

10. Plisson, H. & Beyries, S. Pointes ou outils triangulaires ? Données fonctionnelles dans le Moustérien levantin [suivi des] Commentaires de J. Shea, A. Marks, J-M Geneste et de la réponse des auteurs. *Paléorient* **24**, 5–24 (1998).

11. Pargeter, J. Howiesons Poort segments as hunting weapons: experiments with replicated projectiles. *South African Archaeol. Bull.* **62**, 147–153 (2007).

12. Chesnaux, L. Réflexion sur le microlithisme en France au cours du premier Mésolithique Xe-VIIIe millénaires avant J.-C.: approches technologique, expérimentale et fonctionelle. (Université de Paris 1 Panthéon-Sorbonne, 2014).

13. Geneste, J.-M. & Plisson, H. Technologie fonctionelle des pointes a cran Solutréennes: l’apport des nouvelles données de la grotte de Combe Saunière (Dordogne). in *Feuilles de pierre. Les industries à pointes foliacées du Paléolithique supérieur européen. Actes du colloque international de Cracovie (1989)* (ed. Kozlowski, J. K.) 293–320 (ERAUL, 1990).

14. Caspar, J.-P. & De Bie, M. Preparing for the Hunt in the Late Paleolithic Camp at Rekem, Belgium. *J. F. Archaeol.* **23**, 437–460 (1996).

15. O’Farrell, M. Approche technologique et fonctionnelle des pointes de la Gravette : une analyse archéologique et expérimentale appliquée à la collection de Corbiac (Dordogne). (Université de Bordeaux I, 1996).

16. O’Farrell, M. Les pointes de La Gravette de Corbiac (Dordogne) et considérations sur la chasse au Paléolithique supérieur ancien. in *Approches fonctionnelles en préhistoire, (Actes du XXVe Congrès Préhistorique de France, Nanterre 24-26 novembre 2000)* (eds. Bodu, P. & Constantin, C.) 121–138 (Société préhistorique française, 2004).

17. Sano, K. & Oba, M. Projectile experimentation for identifying hunting methods with replicas of Upper Palaeolithic weaponry from Japan. in *International Conference on Use-Wear Analysis: Use-Wear 2012* (eds. Marreiros, J., Bicho, N. & Gibaja, J. F.) 466–478 (Cambridge Scholars Publishing, 2014).

18. Sano, K. & Oba, M. Backed point experiments for identifying mechanically-delivered armatures. *J. Archaeol. Sci.* **63**, 13–23 (2015).

19. Iovita, R., Schönekess, H., Gaudzinski-Windheuser, S. & Jäger, F. Projectile impact fractures and launching mechanisms: Results of a controlled ballistic experiment using replica Levallois points. *J. Archaeol. Sci.* **48**, (2014).

20. Iovita, R., Schönekeß, H., Gaudzinski-Windheuser, S. & Jäger, F. Identifying weapon delivery systems using macrofracture analysis and fracture propagation velocity: A controlled experiment. *Vertebr. Paleobiol. Paleoanthropology* 13–27 (2016) doi:10.1007/978-94-017-7602-8_2.

21. Neill, L., Clarkson, C. & Schoville, B. Holding your shape: Controlled tip fracture experiments on cast porcelain points. *J. Archaeol. Sci. Reports* **44**, 103505 (2022).

22. Sano, K., Denda, Y. & Oba, M. Experiments in Fracture Patterns and Impact Velocity with Replica Hunting Weapons from Japan. in *Multidisciplinary Approaches to the Study of Stone Age Weaponry* (eds. Iovita, R. & Sano, K.) 29–46 (Springer, 2016).

23. Pargeter, J., Shea, J. J. & Utting, B. Quartz backed tools as arrowheads and hand-cast spearheads: Hunting experiments and macro-fracture analysis. *J. Archaeol. Sci.* **73**, 145–157 (2016).

24. Pargeter, J., Bam, L., De Beer, F. & Lombard, M. Microfocus X-ray tomography as a method for characterising macro-fractures on quartz backed tools. *South African Archaeol. Bull.* **72**, 148–155 (2017).

25. Rots, V. & Plisson, H. Projectiles and the abuse of the use-wear method in a search for impact. *J. Archaeol. Sci.* **48**, 154–165 (2014).

26. Coppe, J., Lepers, C. & Rots, V. Projectiles Under a New Angle: a Ballistic Analysis Provides an Important Building Block to Grasp Paleolithic Weapon Technology. *J. Archaeol. Method Theory* **29**, 1131–1157 (2022).

27. Coppe, J. *et al.* Ballistic Study Tackles Kinetic Energy Values of Palaeolithic Weaponry. *Archaeometry* **61**, 933–956 (2019).

28. Tydgadt, L. & Rots, V. Stick to it! Mechanical performance tests to explore the resilience of prehistoric glues in hafting. *Archaeometry* 1252–1269 (2022) doi:10.1111/arcm.12779.

29. Sano, K. Evidence for the use of the bow-and-arrow technology by the first modern humans in the Japanese islands. *J. Archaeol. Sci. Reports* **10**, 130–141 (2016).

30. Sano, K. *et al.* The earliest evidence for mechanically delivered projectile weapons in Europe. *Nat. Ecol. Evol.* **3**, 1409–1414 (2019).

31. Yaroshevich, A., Kaufman, D. & Marks, A. Weapons in transition: Reappraisal of the origin of complex projectiles in the Levant based on the Boker Tachtit stratigraphic sequence. *J. Archaeol. Sci.* **131**, 105381 (2021).

32. Coppe, J. & Rots, V. Focus on the target. The importance of a transparent fracture terminology for understanding projectile points and projecting modes. *J. Archaeol. Sci. Reports* **12**, 109–123 (2017).

33. Soriano, S. Les microgravettes du Périgordien de Rabier à Lanquais (Dordogne) : analyse technologique fonctionnelle. *Gall. Préhistoire* **40**, 75–94 (1998).

34. Lepers, C. & Rots, V. The important role of bow choice and arrow fletching in projectile experimentation. A ballistic approach. *J. Archaeol. Sci. Reports* **34**, 102613 (2020).

35. Hildred, A., Watson, K., Waller, J. & Hardy, R. *Weapons of Warre: The Armaments of the Mary Rose*. (The Mary Rose Trust, 2011).

36. Strickland, M. & Hardy, R. *The Great Warbow: from Hastings to the Mary Rose*. (Stroud, 2005).

37. Junkmanns, J. *Pfeil und Bogen. Von der Altsteinzeit bis zum Mittelalter*. (Verlag Angelika Hörnig, 2013).

38. Cotterell, B. & Kamminga, J. The Formation of Flakes. *Am. Antiq.* **52**, 675–708 (1987).

39. Tsirk, A. Regarding fracture initiations. in *Lithic Use-Wear Analysis* (ed. Hayden, B.) 83–96 (Academic Press, 1979).

40. Tsirk, A. *Fractures in Knapping*. (Archaeopress, 2014).

41. Amick, D. S. & Mauldin, R. P. Effects of raw material on flake breakage patterns. *Lithic Technol.* **22**, 18–32 (1997).

42. Coppe, J., Taipale, N. & Rots, V. Fracture mechanics and the identification of projectiles: what is the influence of raw material? in *Rock and Roll: 13th International Symposium on Knappable Materials* (eds. Goméz De Soler, B., Soto, M., Chacón, M. G. & Soares Remiseiro, M.) 164 (Adhoc Cultura, 2021).

43. Rots, V. TRAIL: An Experimental Trace and Residue Reference Library for the functional analysis of stone tools in Liège. *OSF Prepr.* 1–8 (2021) doi:10.31219/osf.io/jsak6.

44. Tomasso, S. *et al.* A closer look at an eroded dune landscape: first functional insights into the Federmessergruppen site of Lommel-Maatheide. *Peer Community J.* **1**, e66 (2021).

45. Taipale, N. Hafting as a flexible strategy: variability in stone tool use and hafting at three European Upper Palaeolithic sites. (University of Liège, 2020).

46. Moss, E. H. *The functional analysis of flint implements. Pincevent and Pont d’Ambon: two case studies from the French Final Palaeolithic. BAR International Series 177*. (Archaeopress, 1983).

47. Rots, V. *Prehension and hafting traces on flint tools: a methodology*. (Universitaire Pers Leuven, 2010).

48. Rots, V. Bright spots and the question of hafting. *Anthropol. Praehist.* **113**, 61–72 (2002).

49. Maury, S. Référentiel expérimental : nécessité de méthodes d’enregistrement - Le cas des pointes à cran du programme “technologie fonctionnelle des pointes à cran solutréennes” / Experimental Reference Bases and the Necessity of Recording Methods - The Example of S. *Supplément à la Rev. archéologique du Cent. la Fr.* **47**, 175–184 (2013).

50. Touzé, O. Aux prémices du Gravettien dans le Nord-Ouest européen. Étude de la production des pointes lithiques à Maisières- Canal (province de Hainaut, Belgique). *Bull. la Société préhistorique française* **115**, 455–495 (2018).

51. Jussila, J. Preparing ballistic gelatine - review and proposal for a standard method. *Forensic Sci. Int.* **141**, 91–98 (2004).

52. Gautier, A. Mammifères fossiles. in *La faune du site paléolithique de Maisières-Canal* (eds. Gautier, A., Ballman, P. & de Coninck, J.) 3–20 (Institut royal des sciences naturelles de Belgique, 1973).

53. Lacarrière, J. *et al.* A review of the Gravettian collections from the excavation of Maisières ‘Canal’ (Prov. of Hainaut, Belgium): A combined study of fossil and non-fossil animal resources for alimentary and technical exploitation. in *Les sociétés gravettiennes du Nord-Ouest européen : nouveaux sites, nouvelles données, nouvelles lectures - Gravettian societies in North-western Europe: new sites, new data, new readings. ERAUL 150* (eds. Touzé, O., Goutas, N., Salomon, H. & Noiret, P.) 23–51 (Presses Universitaires de Liège, 2021).

54. Schoch, W. H., Bigga, G., Böhner, U., Richter, P. & Terberger, T. New insights on the wooden weapons from the Paleolithic site of Schöningen. *J. Hum. Evol.* **89**, 214–225 (2015).

55. Thieme, H. Lower Palaeolithic hunting spears from Germany. *Nature* **385**, 807–810 (1997).

56. Touzé, O. D’une tradition à l’autre, les débuts de la période gravettienne. Trajectoire technique des sociétés des chasseurs-cueilleurs d’Europe nord-occidentale. (Université de Liège / Université Paris 1 Panthéon-Sorbonne, 2019).
